# Determining Excited-State Absorption Properties of a Quinoid Flavin by Polarization-Resolved Transient Spectroscopy

**DOI:** 10.1021/acs.jpca.4c01260

**Published:** 2024-05-06

**Authors:** Yi Xu, Martin T. Peschel, Miriam Jänchen, Richard Foja, Golo Storch, Erling Thyrhaug, Regina de Vivie-Riedle, Jürgen Hauer

**Affiliations:** † TUM School of Natural Sciences, Department of Chemistry and Catalysis Research Center, Technical University of Munich, Lichtenbergstraße 4, 85748 Garching, Germany; ‡ Department of Chemistry, Ludwig-Maximilians-Universität München, 81377 München, Germany

## Abstract

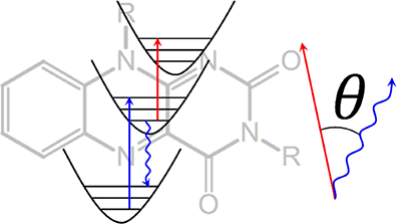

As important naturally occurring chromophores, photophysical/chemical properties of quinoid flavins have been extensively studied both experimentally and theoretically. However, little is known about the transition dipole moment (TDM) orientation of excited-state absorption transitions of these important compounds. This aspect is of high interest in the fields of photocatalysis and quantum control studies. In this work, we employ polarization-associated spectra (PAS) to study the excited-state absorption transitions and the underlying TDM directions of a standard quinoid flavin compound. As compared to transient absorption anisotropy (TAA), an analysis based on PAS not only avoids diverging signals but also retrieves the relative angle for ESA transitions with respect to known TDM directions. Quantum chemical calculations of excited-state properties lead to good agreement with TA signals measured in magic angle configuration. Only when comparing experiment and theory for TAA spectra and PAS, do we find deviations when and only when the S_0_ → S_1_ of flavin is used as a reference. We attribute this to the vibronic coupling of this transition to a dark state. This effect is only observed in the employed polarization-controlled spectroscopy and would have gone unnoticed in conventional TA.

## 1 Introduction

Spectroscopy with polarized light is a versatile tool to disentangle congested regions of molecular absorption spectra and to provide insights into molecular structure. For example, circular dichroism is a standard method to determine the relative content of helical structures in proteins.^1^ Linear dichroism is commonly employed to investigate the structural and orientational properties of molecules in different environments, e.g., to understand the alignment of biomolecules in biological membranes.^2−4^ All polarization-resolved spectroscopic methods rely on the tensorial nature of transition dipole moments (TDMs) connecting initial and final molecular states. For linear and circular dichroism, the initial state is equivalent to the ground state. If the initial state is an electronically excited state, the probed TDM can connect to even higher-lying states, as found in excited-state absorption (ESA) transitions in transient absorption (TA) spectroscopy. Such information on excited-state TDMs is an important benchmark for modern advanced quantum mechanical approaches, such as multireference perturbation theory (CASPT2, NEVPT2), coupled-cluster theory (CC2, CC3, EOM-CC), or double-hybrid time-dependent density functional theory (DH-TDDFT).^5−9^ However, experimental data on excited-state TDM directions are sparse at best. The most common approach relies on TA anisotropy (TAA), which is troubled by diverging signals if the underlying TA signals approach zero.^10,11^ In this work, we employ polarization-associated spectra (PAS) as an extension of TAA, in which the problem of diverging signals is averted. We show how the PAS of a quinoid flavin sample lets us determine the relative TDM angles for all observed ESA transitions with respect to known directions of ground-state TDMs. We also show how transient spectra and TAA can be obtained from quantum chemical simulations using DH-TDDFT and compare the results to the experimental measurements.

Flavins are important naturally occurring chromophores in biology and play essential roles in various metabolic and enzymatic processes in living organisms.^12−14^ They exhibit multiple redox and protonation states,^15^ which allows them to participate in biochemical redox reactions as essential cofactors of many oxidoreductases.^16^ Due to their absorption and emission properties,^17^ flavins also serve as chromophores in several photoreceptors and light-responsive enzymes. These properties make flavins crucial in natural processes, such as DNA repair, cell apoptosis, signal transduction, and biological imaging.^16,18−22^

Photochemically excited flavin cofactors in flavin-dependent enzymes unlock a variety of synthetically interesting catalytic transformations.^23^ In the organic laboratory, molecular flavins serve as versatile and easily modifiable catalysts.^24,25^ Most catalytic applications use quinoid flavins as excited-state oxidants. The corresponding photocatalytic cycle starts with the excitation of the quinoid flavin in its ground state to the singlet excited state (^1^ππ*), followed by an intersystem crossing (ISC) to the photocatalytically active triplet state (^3^ππ*) within several nanoseconds.^25−27^ This efficient ISC process also explains the moderate fluorescence quantum yield of ∼30% at room temperature.^27−29^ The triplet yield can be further enhanced via re-excitation of the initially excited singlet state.^30−32^ The desire to drive such excited-state transitions efficiently underlines the importance of knowing the excited-state TDM directions.

Outside the fields of thermal and photodriven catalysis, Roth et al. studied quantum control systems based on flavins,^33^ in which two nearly identical flavin molecules with subtle differences only in their side chains were excited by a phase-shaped 400 nm pulse. The molecules were distinguished by differences in fluorescence depletion by a subsequent 800 nm pulse. This again calls for an in-depth understanding of the excited-state properties of flavins, in particular the TDMs of flavin’s various excited states.

Despite a large body of work on transient spectroscopy on flavins,^20,34,35^ little is known about the TDM orientation of ESA transitions. Experimental approaches to this problem, based on TAA, have been reported in our earlier work.^36^ Our approach uses TAA data to separate the TA spectra from isotropic samples into linearly independent—and more readily interpreted—spectral components, i.e., PAS.^37−39^ In the present study, we employ this method to dissect the TA signal of a quinoid flavin in solution. Furthermore, we determine the relative angle between the TDMs of two neighboring ESA transitions. We isolate pure ESA signals from ground-state bleach (GSB) and stimulated emission (SE). By comparing to more conventional methods of extracting pure ESA signals, we highlight the advantages of our approach.

## 2 Materials and Methods

### 2.1 Steady-State Spectroscopy

**Figure 1:**
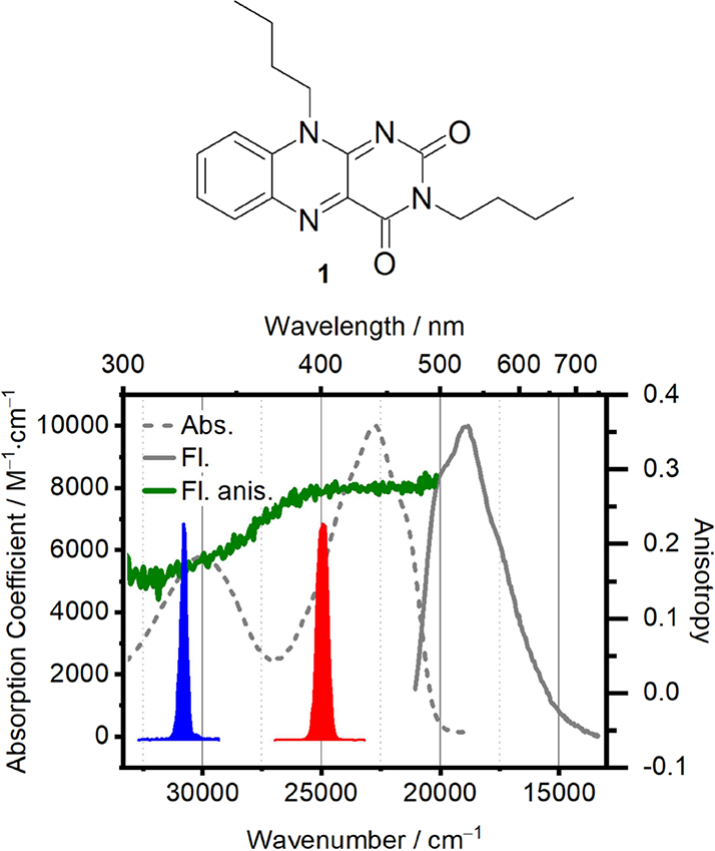
Upper panel shows the molecular structure of (3,10-dibutylbenzo[g]pteridine-2,4(3H,10H)-dione) of quinoid flavin (**1**) in DMF solution. The bottom panel shows the corresponding UV–vis absorption (gray dashed), fluorescence (gray solid, normalized to the strongest absorption peak), and fluorescence anisotropy spectrum (dark green solid). The red (blue) filled areas represent pump pulses at the two employed excitation wavelengths of 400 and 325 nm, respectively.

Linear absorption and emission experiments of the quinoid flavin (3,10-dibutylbenzo[*g*]pteridine-2,4(3*H*,10*H*)-dione) (compound **1**, structure shown in Figure 1) were performed in *N*,*N*-dimethylformamide (DMF) in a 1 cm fused silica cuvette. The absorption spectra were recorded on a PerkinElmer Lambda 365 UV–vis spectrophotometer, while fluorescence and fluorescence anisotropy spectra were measured using an Edinburgh FS5 spectrofluorometer equipped with polarizer assemblies in both excitation and emission paths.

In all fluorescence measurements, the maximum optical density (OD) was kept below 0.05 in order to avoid inner filter effects. The sample for fluorescence anisotropy measurements was prepared in poly(tetrahydrofuran) (poly-THF, average *M*_n_ ≈ 2900, Aldrich chemistry). The polymer sample was kept close to the melting point at 30 °C with an electronic Peltier element in order to simultaneously maintain sufficiently high viscosity for the anisotropy measurements and solvent transparency. The fluorescence (excitation) anisotropy was calculated according to the standard formula as:^40^
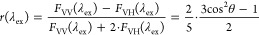
1where *F*_nm_ is the recorded fluorescence intensity, with excitation and emission polarizer settings denoted by subscripts *V* (vertical) and *H* (horizontal). The relative angle θ between the excited and emission TDMs can be retrieved.

### 2.2 TA Spectroscopy

Femtosecond TA experiments were performed using a home-built TA setup detailed previously.^36^ Briefly, a 5 kHz train of 800 nm, 36 fs fwhm laser pulses generated by a regenerative amplifier (Coherent Legend Elite Duo HE+) was divided into pump and probe arms. Two pump wavelengths, 400 and 325 nm, were chosen to selectively excite the S_0_ → S_1_ and S_0_ → S_3_ transitions (see Figure 1). The 400 nm excitation pump pulse was obtained via second harmonic generation (SHG) of the 800 nm fundamental pulse inside a 200 μm-thin beta-barium borate crystal (BBO, Bluebean Optical Tech Ltd.). The 325 nm UV pump is obtained by frequency doubling the output of a tunable noncollinear optical parametric amplifier (NOPA).^41^ The NOPA was used to generate 12 nm pulses full width at half-maximum centered at 650 nm, with a resulting pulse duration of 38 fs as determined by SGH Frequency Resolved Optical Gating (SHG-FROG).^42^ The 400 and 325 nm pump pulse durations were estimated to be 90 and 108 fs (fwhm), respectively, from fitting the instrument response function using the program package OPTIMUS1.^43^ White light for probing was generated by focusing 4 μJ of 800 nm light into a translated 5 mm thick CaF_2_ crystal. The white light was split into a probe part and a reference part. The probe pulse was focused into the sample by using a 150 mm focal length spherical mirror and the spot size is 30 μm. The probe spatially overlaps with the center of the pump and is recollimated after the sample by using an achromatic lens with a 75 mm focal length. The probe and reference laser pulses were detected using a home-built prism spectrometer in combination with a pair of high-speed CMOS linear array cameras (Glaz LineScan-I-Gen2, Synertronic Designs). All the measurements were performed in DMF solution at an optical density (OD) of 0.1 at the absorption maxima in a flow cuvette with 100 μm optical path length. For both 400 and 325 nm pump–probe measurements, the experiments were done by setting the pump and probe pulses orthogonal, parallel, and at a magic angle (MA) with each other by using a waveplate in the pump arm (300–470 nm λ/2 plate, B.Halle Nachfl. GmbH). Sketches of the 400 and 325 nm pump–probe setups can be found in Figures S2 and S3.

### 2.3 TA Anisotropy and PAS Calculation

TA spectra were recorded with excitation- and probe-pulse polarizations being either parallel (*S*_∥_(λ, *t*)) or perpendicular (*S*_⊥_(λ, *t*)) to each other. The TAA can be calculated as:^44−46^
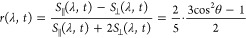
2where θ is the angle between the excited and detected TDMs. In analogy with the fluorescence anisotropy, *r*(λ, *t*) provides useful structural information as it reports on relative angles between the initially excited and probed TDMs.^11^ However, the interpretation of TAA data is less straightforward, in particular because the signal diverges whenever the denominator in eq 2, equal to three times the MA signal amplitude, approaches zero. PAS mitigates this problem.^36,47,48^ PAS are projections of the isotropic TA spectrum into contributions that are either parallel (*S*_*z*_) or orthogonal (*S*_*y*_) to the TDM initially excited by the pump pulse. The expressions for these projections are given by
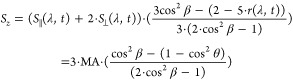
3and
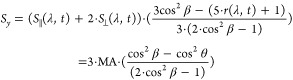
4

The terms  and  are analogous to *r*(λ, *t*), in which they report on the angle θ between pumped and probed TDMs, but are expressed in the frame of the molecule. The detailed derivation of the above expressions can be found elsewhere.^28^ The coordinate system of the molecular frame is defined by the direction of the initially excited TDM, by convention set to be parallel to the *z*-axis. In eqs 3 and 4, angle β is introduced. β rotates the molecular-frame *z*-axis away from the originally excited TDM. Accordingly, setting β to zero leaves the initially excited TDM parallel to the *z*-axis and recovers the expressions used in earlier works.^47−49^ Conceptually, this rotation by the angle β is useful as it alters how strongly the signal at a given wavelength projects into the PAS components given by eqs 3 and 4. For a spectrally isolated feature, the signal strength is minimized (maximized) in one PAS component exactly when β equals the angle θ between the initially excited TDM and the TDM being probed at the given wavelength. Importantly, the *S*_*z*_/*S*_*y*_-PAS will be free of diverging signals often encountered in TAA when the TA spectra show a zero-crossing (see eq 2 and ref (36)).

### 2.4 Quantum Chemical Simulations and Modeling of the Spectra

Modeling EAS is a challenging task since it requires an accurate description of high-lying excited states. Thus, butyl side chains of the quinoid flavin **1** as depicted in Figure 1 were replaced by methyl groups to avoid extensive sampling due to high conformational flexibility. This change was found not to alter the electronic structure of **1** significantly. TDDFT using the range-separated hybrid functional ωB97-X-D3 was not able to predict bright states at the correct energy to explain the observed ESA features. However, using a range-separated double hybrid functional (SCS-ωPBEPP86),^50^ bright states close to the observed ESA features were obtained. At the Franck–Condon point, these states match calculations at the DLPNO-STEOM-CCSD level. However, a solvent model for excited states was necessary to obtain the correct Stokes shift. As there is, currently, no implementation of such a model for DLPNO-STEOM-CCSD, this method could not be used for calculations away from the Franck–Condon point. Further discussions are hence based on calculations at the SCS-ωPBEPP86 level.

All quantum chemical calculations were performed using the ORCA 5.0 program package.^51^ Minima of the ground state (S_0_) and first excited state (S_1_) were optimized using ωB97-X-D3/ma-def2-TZVP.^52^ Excitation and emission energies at these points were calculated using the range-separated double-hybrid functional SCS-ωPBEPP86^50^ with the aug-cc-PVTZ basis set. All calculations used a linear-response conductor-like polarizable continuum model (LR-CPCM) solvent model with DMF as the solvent. ESA and SE transitions are calculated at the optimized S_1_ structure with the solvent model equilibrated to the first excited state. GSB transitions are calculated at the optimized S_0_ structure with the solvent equilibrated to S_0_. Detailed inputs to perform the calculations can be found in the Supporting Information.

Vibrational broadening was included in the calculated spectra on a semiclassical level. To this end, 250 samples were drawn from a Wigner distribution^53,54^ centered at each of the optimized structures and excitation and/or emission energies for each sample were calculated. With these data in hand, a fit to the TA traces at 1 ps using three parameters (Gaussian width, redshift, relative emission intensity) was performed to model the experimental data. Accordingly, the excitation and emission energies from the Wigner distribution were convoluted with a Gaussian (σ = 0.093 eV), red-shifted by 0.110 eV, and emission intensities were scaled relative to absorption by a factor of 0.56. Finally, all absorption/emission intensities were scaled to match the respective intensities of the experimental MA spectra. Further details on the calculation of spectra for parallel and perpendicular polarizations of the pump and probe pulses can be found in the Supporting Information.

## 3 Results and Discussion

### 3.1 Absorption and Fluorescence Spectroscopy

A simplified flavin chromophore was chosen for this study in order to obtain experimental data as close as possible to the calculated data. The *N*3,*N*10-disubstituted flavin **1** serves this purpose, while the two butyl substituents ensure solubility. We show the steady-state absorption (gray dashed line) and emission spectra (gray solid line) of DMF solutions of **1** in TDM representation^55,56^ in Figure 1, in which we correct the absorption spectrum by dividing the first power of the wavenumber and the emission spectrum by the third.^55^ The absorption spectrum of **1** exhibits two broad absorption features in the UV/vis range, with peaks at 440 nm (22,727 cm^–1^, S_0_ → S_1_) and 330 nm (30,303 cm^–1^, S_0_ → S_3_). There is evidence for an optically dark *n*π* state (S_2_) between these two bands.^27,29,35^ The shape and central wavelength of the two absorption peaks are in agreement with previous works.^34,57−60^ The determined molar extinction coefficients of 10,000 and 8000 M^–1^ cm^–1^ are in good agreement with literature values for flavin in aprotic solvents.^34,61^ The 1300 cm^–1^ vibronic progression observed in both absorption and emission corresponds largely to C–C and C–N stretching and bending vibrations of the isoalloxazine ring.^62^ The fluorescence spectrum peaks around 530 nm (18,868 cm^–1^) and shows good mirror image symmetry with the absorption.

**Figure 2:**
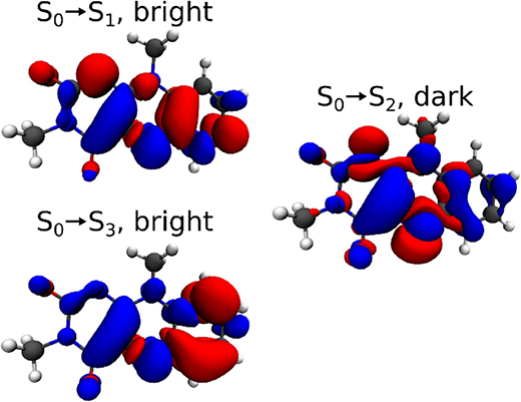
Density differences for the relevant to the static absorption spectrum, GSB, and SE transitions of the quinoid flavin (isovalue: 0.001) were calculated using ωPBEPP86^50^/aug-cc-PVTZ. Electron density is shown in red, and hole density is shown in blue.

Thus, the lowest energy absorption and emission involve the same electronic state, S_1_. Figure 2 shows the density difference associated with the transitions in the observed spectral range. The S_0_ → S_1_ transition is associated with a redistribution of electrons inside the π system along the long axis of the molecule. The third excited singlet state, S_3_, is also of ππ* character and forms the second band centered at 330 nm. In contrast, S_2_(*n*π*) is symmetry forbidden, as it redistributes electron density from the π system toward the oxygen lone pairs outside the ring (σ symmetry). However, the vibronic coupling of this state to its neighbors likely influences the observed line shapes.^29,35^

The excitation fluorescence anisotropy of **1**, calculated according to eq 1, is superimposed on the spectra in Figure 1 as a dark green solid line. *r*(λ) reaches a maximal value of ∼0.28 at the red edge of the absorption spectrum. This failure to reach the limiting value of 0.4 implies substantial rotation of the TDM upon excitation, resulting in an effective angle of ∼27° between the relevant TDMs for absorption and emission.^29^ The anisotropy of the S_0_ → S_3_ transition at 330 nm is similarly low, reaching a value of only 0.15—corresponding to an effective angle θ of 40°. We thus determine the angle between S_0_ → S_1_ and S_0_ → S_3_ to 13°, in good agreement with the 20 ± 5° value from earlier theoretical^63^ and experimental studies.^29^

### 3.2 Magic Angle TA Spectroscopy: Experiment and Simulation

**Figure 3:**
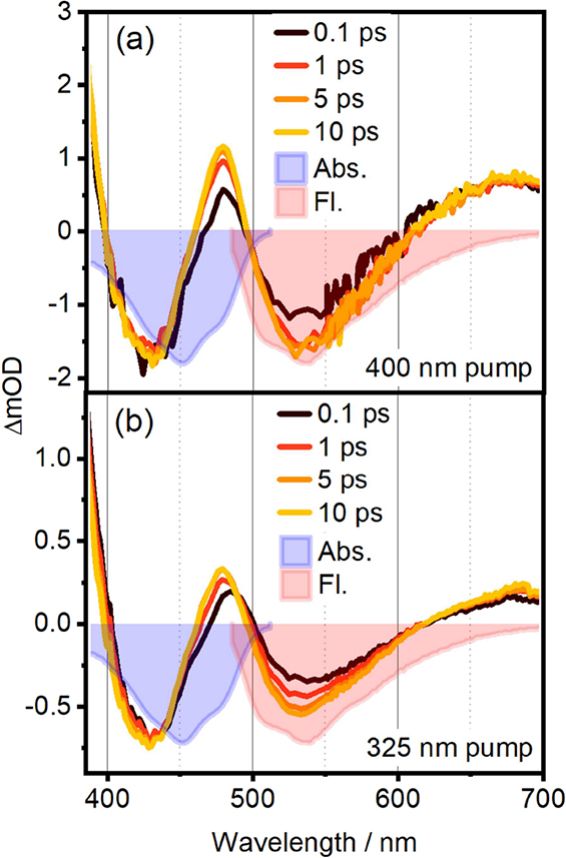
TA spectra slices at 0.5, 1, 5, and 10 ps for (a) 400 and (b) 325 nm pump. The light blue (light red) filled curves are the absorption/fluorescence spectra of quinoid flavin with inverted signs.

In Figure 3, we show selected MA TA spectra of **1** after excitation at 400 (panel a) and 325 nm (panel b). The spectral structure and dynamics observed here are in good agreement with previously reported results.^60,64^ For both employed excitation wavelengths, the negative signals at 440 and 530 nm can be attributed to GSB and SE, respectively. Inverted absorption (light blue-filled) and fluorescence (light red-filled) spectra are shown for comparison. Positive-valued ESA features overlap with the GSB and SE over most of the UV/vis spectral range, resulting in three distinct positive bands: sub-400 nm, at 478 nm, and above 600 nm. We refer to these as UV-ESA, blue-ESA, and red-ESA in the following but exclude the UV-ESA from further discussion, as we only detect its red edge. The dynamics after 400 and 325 nm excitation are compared in Table 1. Employing a global kinetic analysis scheme,^65^ we obtain three lifetime components (see Table 1, the corresponding evolution-associated spectra are shown in Figure S5). We note that the overall shape and dynamics of the TA spectra for 325 nm excitation (excitation to S_3_) and 400 nm excitation (excitation to S_1_) are similar and the deactivation dynamics show no relevant dependence on excitation wavelength. Thus, to explain the observed similarities there has to be ultrafast internal conversion between S_3_ and S_1_, likely mediated by the dark S_2_ state on a few-femtosecond time scale. Accordingly, conical intersections between S_3_ and S_2_ as well as S_2_ and S_1_ were found close to the Franck–Condon region (see Supporting Information Sections 8–11). The observed components with lifetimes τ_1_ and τ_2_ are associated with structural relaxation and -most likely- solvation dynamics of S_1_. The shorter lifetime for this component after 325 nm excitation is explained by the increased excess energy after internal conversion to S_1_.^35,57,66^ The long-lived component τ_3_ is consistent with the fluorescence lifetime of **1**, which was determined to be 5.7 ns in an independent time-correlated single-photon counting measurement.

**Table 1:** Time Constants for **1** Extracted by Global Lifetime Analysis of MA TA Spectra Pumped by 400 and 325 nm (Evolution-Associated Spectra Are Shown in Figure S5a,b)^a^

	400 nm	325 nm
τ_1_	200 fs	170 fs
τ_2_	19.5 ps	4.7 ps
τ_3_	+∞	+∞

a The long-lived component τ_3_ stems from the 5.7 ns fluorescence lifetime of **1**, which is not resolved in the TA delay time window.

**Figure 4:**
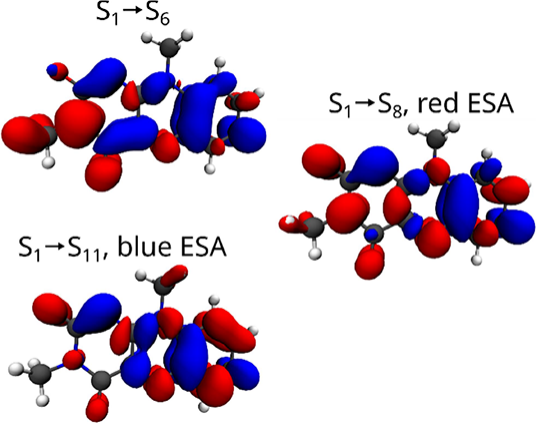
Bright transitions contributing to the ESA with nomenclature (“red ESA”, “blue ESA”) from Section 3.2. See Figure 2 legend and associated discussion for details.

**Figure 5:**
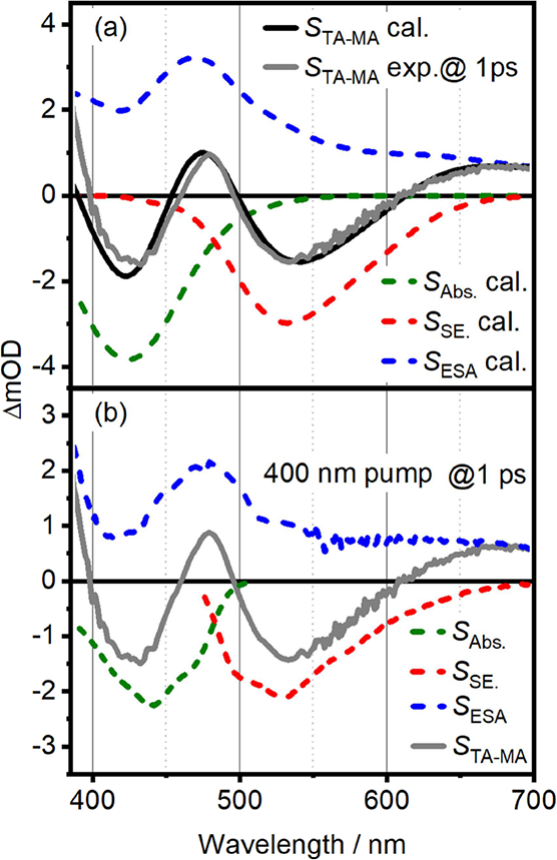
(a) TA slice at 1 ps for 400 nm pump (gray), compared to calculated TA (black). The dashed lines indicate the GSB (dark green), SE (red), and ESA (blue) contributions to the calculated signal. The fit of the calculated signal to the experimental data is discussed in the main text. (b) Pure ESA spectrum (blue dashed) calculated according to eq 5. The components entering eq 5 are the total TA signal (gray solid) at 1 ps delay time, and the scaled absorption (dark green dashed) and SE spectrum (red dashed).

Figure 4 illustrates the density differences associated with the ESA transitions. The numbering of the final states depends significantly on the quantum chemical method and the geometry. The state-ordering is presented here for SCS-ωPBEPP86/aug-cc-PVTZ at the S_1_ minimum, the characters of the bright states are, however, consistent with calculations at the DLPNO-STEOM-CCSD level of theory. Due to symmetry, three states of ππ* character form the main contribution to the observed ESA signal. The S_1_ → S_6_ and S_1_ → S_8_ transitions contribute to the red ESA and the S_1_ → S_11_ transition to the blue ESA. Of these transitions, S_1_ → S_11_ and S_1_ → S_6_ are polarized along the long axis of the molecule, while S_1_ → S_8_ is polarized along the short axis of the molecule. The full MA signal calculated from the Wigner sampling is displayed in Figure 5a, together with the experimental signal at 1 ps.^53,54^ The overall agreement of the calculated and measured signals is excellent. The maximum of the ESA (blue line) in the blue region is due to the S_1_ → S_11_ transition which has the highest oscillator strength of all the ESA transitions. S_1_ → S_6_ and S_1_ → S_8_ form a tail toward the red edge of the spectrum that overlaps with the SE and forms the red ESA band. As the Wigner distribution contains nonsymmetric structures, many other states also contribute, leading to a broad total ESA signal.

To validate the simulation results, we employ a commonly used approach to isolate pure ESA spectra. We apply the following expression:
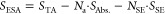
5where *S*_ESA_, *S*_Abs._, *S*_SE_, and *S*_TA_ are the pure ESA spectrum, absorption spectrum, SE spectrum—approximated as the fluorescence spectrum in the TDM representation—and TA spectrum, respectively. *N*_a_ and *N*_SE_ represent scaling factors for GSB and SE, respectively, *N*_a_ is obtained via the calculated excitation probability, defined by the pump fluence and the overlap between the pump and absorption spectrum.^67^ The ratio of *N*_a_ and *N*_SE_ was set to 1.07 to ensure that the pure ESA signal at 560 nm is positive.

The extracted pure ESA spectrum at 1 ps pump–probe delay, and 400 nm excitation is shown as the gray solid line in Figure 5b. The according spectra for 325 nm pumping (not shown) are qualitatively similar. The ESA spectrum exhibits only positive values as expected and consists of a shoulder below 400 nm (UV-ESA), a peak centered at 480 nm (blue-ESA), and a broad red feature above 610 nm (red-ESA). As evident from the discussion of eq 5, the most questionable assumption behind this approach is equating the SE spectrum with the fluorescence spectrum in TDM representation. Any SE from unrelaxed excited states will lead to uncertainties in the obtained ESA spectra. Furthermore, no information on the relative angle between the observed excited-state transitions can be obtained. Anisotropy-based approaches provide elegant solutions to these problems. Takaya et al.^68^ resolved the spectral components for two overlapping species by applying eqs S1 and S2 (see Supporting Information) to isolate two components with different initial anisotropy values (see Figure S6).

As apparent from Figure 5, the qualitative agreement between experiment and simulation is very good with respect to pure ESA features, considering the uncertainties discussed in the previous section. In an effort to arrive at a more quantitative comparison for excited-state properties, we resort to relative angles between TDMs. An established way of getting to this information is measuring TAA spectra as discussed in the next section. The relation between anisotropy and relative angles is again given by the standard expression (see eq 2).

### 3.3 TA Anisotropy

**Figure 6:**
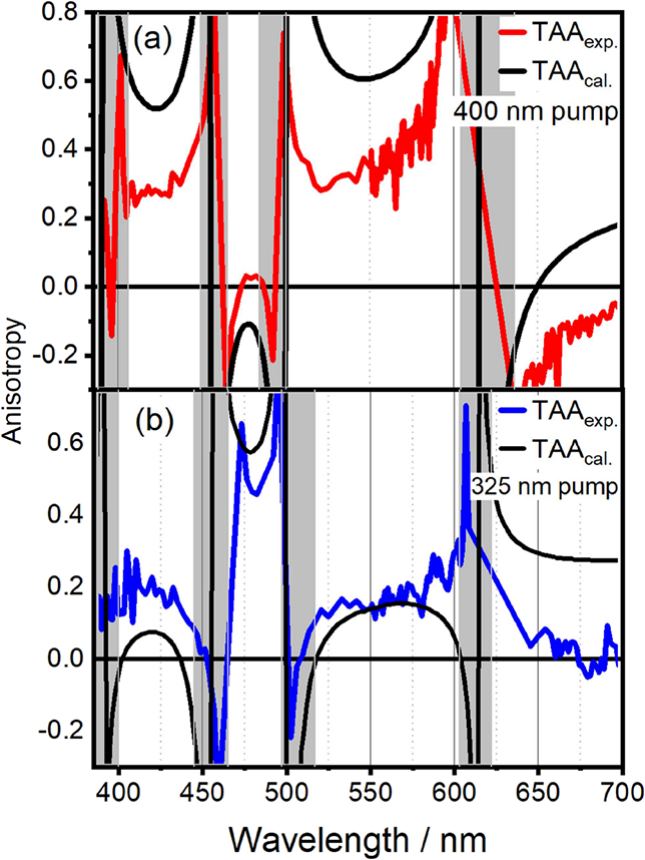
TA anisotropies of **1** with (a) 400 nm pump and (b) 325 nm pump. Experimental anisotropies are shown in red for 400 nm excitation and blue for 325 nm excitation, calculated anisotropies are shown in black. Experimental anisotropies are given for a pump–probe delay of 1 ps.

In Figure 6a,b, we show TAA slices at 1 ps after excitation at 400 (red full line) and 325 nm (blue), respectively. These spectra represent the relation between transition dipoles well since diffusional depolarization is minimal at such short time scales (see Figure S4). The spectra diverge at wavelengths of zero-crossings in the TA spectrum, indicated by the gray-shaded area. The TAA spectra for 400 and 325 nm excitation are offset from each other due to their initially excited TDMs being nonparallel (see Figure 1). Quantitatively, there are significant differences between the calculated and measured anisotropies. As illustrated by a comparison between Figure 6a,b, the simulated anisotropies are much closer to the experiment in the case of 325 nm excitation. As the only difference in the simulation of these two spectra is the TDM directions of the absorbing states S_1_ and S_3_ at the Franck–Condon point, there has to be a source of error that predominantly affects S_1_ in the Franck–Condon region.^66^ Due to the presence of an S_1_–S_2_ conical intersection in the Franck–Condon region, excitation at 400 nm leads to a wavepacket composed of vibronic states that are a mixture of S_1_(ππ*) and the dark S_2_(*n*π*).^66^ This mixing apparently alters significantly the TDM direction. These vibronic coupling effects are not reproduced without an explicit treatment of the excited-state dynamics of the system.^53,54^ Accordingly, our model fails to reproduce the depolarization of the S_1_ state (see also Figure 1, green line), i.e., the 27° rotation between the absorbing and emitting dipoles. Two other factors should also be considered: first, there are known, significant solvent influences on the excited states of flavins,^35,69,70^ which also show up in our simulations as an inability to capture the Stokes shift without an appropriate solvent model. Changes in nontrivial solvent interactions that are not captured by a continuum model might lead to a rotation of the effective transition dipole moment of a solvent–solute complex upon relaxation. Such a mechanism has been proposed previously by Weigel et al.,^66^ who suggest that solvent rearrangement is coupled to *n*π*/ππ* interconversion. Finally, for the DH-TDDFT functional used in this work, energies are expected to be of higher accuracy than TDMs. Especially, the CIS(D) correction used in the double-hybrid part of the functional applies only to the excitation energies; TDMs are only of hybrid TDDFT quality.

We note that the above discussion on the possible role of vibronic coupling in S_1_ would not have been possible based on the simulation of TA spectra alone (see Figure 5), where the agreement between measured data and simulation was satisfactory. Only the analysis of TAA data in Figure 6 reveals the underlying discrepancy between the experiment and TDDFT-based theory. To investigate this matter further based on an easily quantifiable observable, we now turn to the relative angle between TDMs. The latter can in principle be calculated from TAA data using eq 2. However, this approach requires separated spectral components; if at a given detection wavelength two or more spectral species overlap, their values for *r*(λ, *t*) will add up. This problem is addressed by using PAS in the subsequent section.

### 3.4 Polarization-Associated Spectra

**Figure 7:**
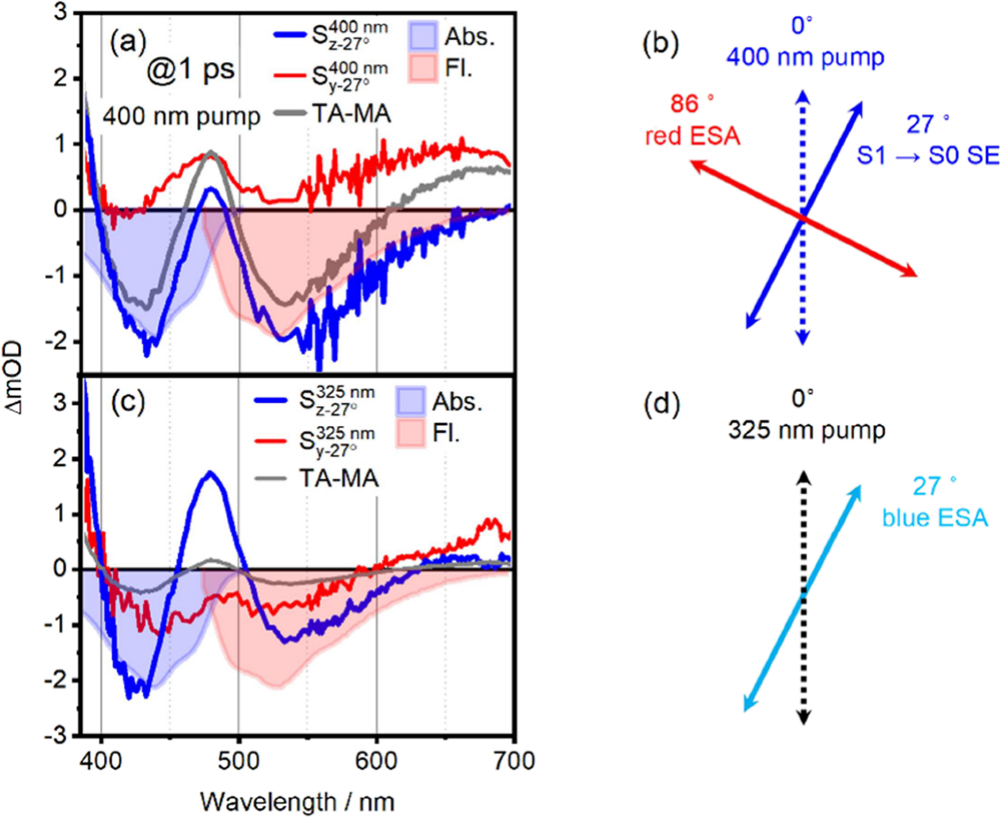
(a) PAS for 400 nm excitation. The *S*_*z*–27°_^400 nm^ component (blue) optimizes the SE component with respect to the MA TA spectrum (dark gray). Panel (b) sketches the resulting TDM directions. (c) PAS for 325 nm excitation and β = 27°, emphasizing the blue ESA peaking at 480 nm. The resulting TDM directions are sketched in (d).

In the following section, we will show that PAS delivers the angle of the TDM directions by increasing β. The first step in the PAS approach is to calculate *S*_*z*_ and *S*_*y*_ according to eqs 3 and 4 with coordinate-system rotation angle β set to zero. In the case of **1** studied here, we found poor separation in PAS for both pump wavelengths, with essentially all signals contributing in both PAS components (see Figure S7). In other words: for β = 0, there are no spectral features that would be minimized (maximized) in neither *S*_*z*_ nor *S*_*y*_, meaning that the PAS approach does not bring new insights for the chosen value of β = 0. The fluorescence anisotropy data in Figure 1 already imply an angle θ = 27° between excitation and emission TDM as a useful starting point for β. Setting β = θ = 27° for 400 nm pump wavelength should hence maximize SE-contributions in *S*_*z*–27°_^400 nm^, where the superscript denotes the pump wavelength. The corresponding PAS is shown in Figure 7a, blue line. We see a clear enhancement of both GSB and SE contributions in *S*_*z*–27°_^400 nm^ compared to TA-MA, see blue vs gray line in Figure 7a. We note that PAS exhibit smooth signals over a broad detection range, which is an important advantage of TAA and its diverging signals (see Figure 5). This means that PAS allows for global (target) analysis (GTA), shown for PAS and TA-MA in Figure S5.

For easier identification of GSB and SE signals, we add the absorption (blue-filled area) and fluorescence spectrum (red-filled area). The fact that the GSB signal can be enhanced by a change of β implies that there is an ESA feature at the same detection wavelength but with a different TDM direction. Accordingly, the *S*_*y*–27°_^400 nm^ PAS (red line) shows ESA signals in the spectral region where *S*_*z*–27°_^400 nm^ exceeds the TA-MA signal. We note that *S*_*y*–27°_^400 nm^ is positive throughout the entire detection window. Between 380 and 530 nm, there is good overlap between the pure ESA signal and *S*_*y*–27°_^400 nm^. The pure ESA signal is discussed in Figure 5 and shown as a dotted line in Figure 7a. The red-ESA for detection wavelengths longer than 530 nm is enhanced beyond the level of the pure ESA signal in *S*_*y*–27°_^400 nm^. Furthermore, the red-ESA is completely missing in *S*_*z*–27°_^400 nm^, as this component overlaps perfectly with the fluorescence spectrum between 530 and 700 nm (see Figure 7a, red filled area). This leads us to the conclusion that the TDM of the red-ESA must be nearly orthogonal to the TDM of the SE. The latter is at 27° with respect to the ground-state transition at 400 nm, as determined by fluorescence anisotropy, see Figure 1. We summarize these findings pictorially in Figure 7b. As compared to TAA and its diverging signals (see Figure 6).

We found β = 31° is the best-suited value for minimizing the red-ESA in *S*_*z*–31°_^400 nm^ (see Figure S9a), which gives us an 86° angle between the TDM of the red-ESA and SE. The blue-ESA transition, peaking at 480 nm, does not lend itself to such a straightforward assignment, as it is observable both in *S*_*z*–27°_^400 nm^ and *S*_*y*–27°_^400 nm^. Given the strong spectral congestion in this wavelength range, we refrain from determining the TDM angle of the blue-ESA via its relative contribution to *S*_*z*–27°_^400 nm^ and *S*_*y*–27°_^400 nm^. Instead, we find the strongest enhancement of the blue-ESA signal at β = 27° after 325 nm excitation. The respective spectra are shown in Figure 7c (We note that the correspondence between the values of β in Figure 7a,c is coincidental). The *S*_*z*–27°_^325 nm^ PAS, shown in blue in Figure 7c, shows strong enhancement of the blue-ESA peaking at 480 nm in comparison to the TA-MA spectrum. More importantly, the corresponding *S*_*y*–27°_^325 nm^ PAS lacks any ESA feature at 480 nm, as *S*_*y*–27°_^325 nm^ shows good overlap between the absorption and SE spectrum in this region (see red vs filled blue and red curve). Hence, we can conclude that the blue-ESA’s TDM has a 27° angle with respect to the TDM of the 325 nm transition. This scenario is depicted in Figure 7d.

Knowing the angle between the TDM of S_0_ → S_3_ (325 nm) and the TDM of S_0_ → S_1_ (400 nm) to be 13° (see Figure 1), we can deduce the angle between the TDMs of the 400 nm transition and the TDM of the blue-ESA to be 13° + 27° = 40°. An angle so close to 45°, where the PAS components in eqs 5 and 6 become degenerate, explains why we were unable to find a value for β to maximize the blue-ESA while minimizing GSB or SE signals. The red-ESA can be minimized when β = 35°, as can be seen in *S*_*y*–35°_^325 nm^ at 700 nm (see Figure S9b). This indicates that the angle between the red-ESA and the S_0_ → S_3_ TDMs is 55°. The comparison of angles between TDMs obtained experimentally and theoretically are listed in Table 2.

**Table 2:** Comparison of Angles between Each Transition Obtained by Experimental Method and Theoretical Calculation

TDM_1_	TDM_2_	experimental method	θ_1–2_ exp.	θ_1–2_ calc.
S_0_ → S_3_	S_0_ → S_1_	fl. anis. and 325/400 nm pumped PAS	13°	33°
S_1_ → S_0_ (SE)	S_1_ → S_8_ (red ESA)	400 nm pumped PAS	86**°**	83°
S_0_ → S_3_	S_1_ → S_0_ (SE)	fl. anis in combination with 400 nm pumped PAS	40°	35°
S_0_ → S_3_	S_1_ → S_11_ (blue-ESA)	325 nm pumped PAS	27°	4.9°
S_0_ → S_3_	S_1_ → S_8_ (red-ESA)	325 nm pumped PAS	55°	62°

The theoretically calculated angles between S_1_ → S_0_ (SE) and S_1_ → S_8_ (red ESA), S_0_ → S_3_ and S_1_ → S_0_, as well as S_0_ → S_3_ and S_1_ → S_8_ are 83, 35, and 62°, respectively, which are in good agreement with our experimental results. However, there are nonnegligible discrepancies (20°) in the angles when comes to S_0_ → S_1_ and S_1_ → S_11_ (blue-ESA). The deviation concerning S_0_ → S_1_, as we mentioned in 3.1, can be explained by vibronic coupling of S_1_ to the neighbor dark state S_2_. Similarly, the presence of dark states energetically close to S_11_ can also influence the TDM orientation of S_1_ → S_11_. Furthermore, the relatively high energy of the S_11_ state (4.92 eV or 39,683 cm^–1^) can also be the source of the observed inaccuracies of TDM calculations.

## 4 Conclusions

We employ polarization-resolved transient spectroscopy to reveal new insights into quinoid flavin. While there is good agreement between magic angle TA and simulations (see Figure 5), polarization-resolved spectra show intriguing differences. We explain them by vibronic coupling between the S_1_ state and the optically dark S_2_ state. As a result, all TDMs involving the S_1_ state deviate from experimental results, while there is a good agreement for all other transitions, as summarized in Table 2. In other words, our polarization-resolved approach highlights the influence of vibronic coupling, which would have remained hidden in conventional TA spectroscopy. This makes polarization-controlled spectroscopy an insightful method to benchmark the prediction of transition dipole moment directions by quantum chemical methods for electronic excited states. On a broader scope, our findings have the potential to facilitate and inspire advanced photocatalytic strategies, in which the re-excitation of excited states increases the catalyst’s redox potential.^71^ Knowing the relative angle between TDMs will allow for optimizing the yield of the re-excitation process.

## Supporting Information Available

Experimental details, evolution-associated spectra, Red-ESA disentanglement, transient anisotropy spectra time traces, PAS for β = 0°, minimizing Red-ESA, calculation of polarized spectra and anisotropies, ORCA 5.0 inputs, coordinates of optimized structures of (3,10-dimethylbenzo[*g*]pteridine-2,4(3*H*,10*H*)-dione, calculated (transient) absorption energies and transition dipoles at the minima (PDF)
