# Supplementary material for: Determining Excited-State Absorption Properties of a Quinoid Flavin by Polarization-Resolved Transient Spectroscopy
Source: J Phys Chem A. 2024 May 6;128(19):3830–9. doi: 10.1021/acs.jpca.4c01260 (PMC11103687; doi:10.1021/acs.jpca.4c01260)
Supplement: Supplementary file 1 — jp4c01260_si_001.pdf [file jp4c01260_si_001.pdf]

Supporting Information for

Determining Excited State Absorption Properties of a Quinoid Flavin by Polarization-Resolved Transient Spectroscopy

Yi Xu[1], Martin Peschel[2], Miriam Jänchen[1], Richard Foja[1], Golo Storch[1], Erling Thyrhaug[1], Regina de Vivie-Riedle[2] and Jürgen Hauer[1]*

[1]Technical University of Munich, TUM School of Natural Sciences, Department of Chemistry and Catalysis Research Center, Lichtenbergstraße 4, 85748 Garching, Germany;

[2]Ludwig-Maximilians-Universität München, Department of Chemistry, 81377 München, Germany;

Table of Contents

1. Experimental Section

2. Transient Anisotropy Spectra Time Traces

4. Evolution Associated Spectra

5. Red-ESA disentanglement

6. PAS for $\beta = 0°$ and $\beta = 40°$

7. Minimizing Red-ESA

8. Calculation of Palarized Spectra and Anisotropies

9. ORCA[8] Inputs

10. Coordinates of Optimized Structures of 3,10-Dimethylbenzo[*g*]pteridine-2,4(3*H*,10*H*)-dione

11. Calculated (Transient-) Absorption Energies and Transition Dipoles at the Minima

1. Experimental Section

1.1. General Remarks

1.1.1. Synthetic Techniques, Solvents, and Chemicals

Room temperature is defined as 21–23°C. All reactions with air-sensitive reactants were carried out under an argon atmosphere (Ar 4.8) applying standard Schlenk technique. Unless otherwise noted, all chemicals were obtained from Sigma-Aldrich, Acros, TCI, abcr, or Alfa Aesar and used without further purification. Dichloromethane (2×MB-KOL-A type 2, aluminium oxide), diethyl ether (1×MB-KOL-A type 2, aluminium oxide), and tetrahydrofuran (2×MB-KOL-M type 2, 3 Å molecular sieves) were obtained from a MBSPS 800 MBraun solvent purification system. All other solvents were purchased from commercial suppliers and used without further purification unless otherwise noted. *N*,*N*'-dimethyl formamide (DMF) for spectroscopy was purchased from Sigma-Aldrich (HPLC grade ≥ 99 %).

1.1.2. NMR Spectroscopy

NMR spectra were recorded on Bruker AVHD-300, AVHD-400, AVHD-500, or AV-III-500 spectrometers at ambient temperature unless otherwise noted. Deuterated NMR solvents were purchased from Deutero GmbH or Sigma Aldrich and were used without further purification. Spectra were processed with MestReNova 10.0.1 using the manual phasing and polynomial baseline correction capabilities. Splitting was determined using the multiplet analysis function with manual intervention as necessary. Spectral data is reported as follows: chemical shift (multiplicity [singlet (s), doublet (d), triplet (t), quartet (q), pentet (p), multiplet (m)], coupling constant, integration, assignment). Broad signals are labeled as such (br). Chemical shifts are reported in ppm (δ) and coupling constants are reported in Hz. Resonances are referenced to solvent residual signals.[1] Assignment of all signals was performed by two-dimensional experiments (^{1}H-^{1}H-COSY, ^{1}H-^{13}C-HSQC-ME, and ^{1}H-^{13}C-HMBC). In cases where an unambiguous assignment was not possible, this is indicated by "/" between atom positions in question, while "," is used in cases where resonances of two or more atoms overlap. Apparent

multiplets which occur as a result of accidental equality of coupling constants to those of magnetically non-equivalent protons are marked as virtual (*virt.*).

1.1.3. Mass spectrometry

High-resolution mass spectrometry (HR-ESI) was performed on a Thermo Scientific LTQ-FT Ultra (ESI) instrument.

1.1.4. Infrared Spectroscopy

Infrared spectra were recorded on a Perkin Elmer Frontier ATR/FT-IR spectrometer, and ν_{max} are partially reported in cm^{-1}.

1.1.5 Liquid Chromatography

Analytical HPLC measurements were performed on Thermo Fisher Ultimate 3000 series instruments equipped with DAD 3000 detectors, LPG 3400SD pumps, TCC 3000SD thermostats, and WPS 3000SL ANALITICAL autosamplers.

Analytical thin layer chromatography was performed using 60 Å Silica Gel F254 (Merck) pre-coated glass plates. TLC plates were visualized by irradiation with a UV lamp or staining. Preparative flash column chromatography was performed on silica 60 (Merck, 230-400 mesh).

1.2. Flavin Synthesis

1.2.1. *N*-Butyl-2-nitroaniline (SI-1)

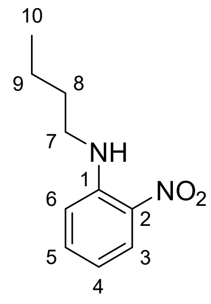

SI-1

Under inert conditions, 2-fluoronitrobenzene (3.73 mL, 5.00 g, 35.4 mmol, 1.00 equiv.) was dissolved in pyridine (35.4 mL, 1.00 M). *n*-Butylamine (3.50 mL, 2.59 g, 35.4 mmol, 1.00 equiv.) and potassium carbonate were added. The suspension was stirred at 80 °C for 16 h. After cooling to room temperature, CH_2Cl_2 (500 mL) was added and the solids were removed to obtain an orange solution. The solvent was removed *in vacuo* and co-evaporated with toluene was performed until the pyridine was fully removed (approx. 4×15 mL toluene) to afford SI-**1** as an orange solid (6.88 g, >99 %). No further purification was performed and the crude product was used for the subsequent step.

1.2.2. *N*[1]-Butylbenzene-1,2-diamine (SI-2)

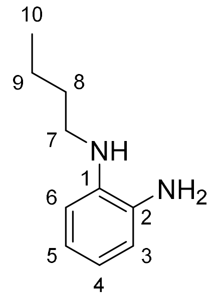

SI-2

Under inert conditions, aniline SI-**1** (6.88 g, 35.4 mmol, 1.00 equiv.) was dissolved in acetic acid (35.4 mL, 1.00 M) before adding palladium on charcoal (10 %, 1.13 g, 1.06 mmol, 0.03 equiv.). The orange solution was degassed by freeze-pump-thaw (3x) before adding a balloon filled with hydrogen. The solution was stirred under an atmosphere of hydrogen for

16 h until full consumption of the starting material. The catalyst was separated by Whatman® filtration into a separate flask under inert conditions affording a faint orange solution of diamine SI-**2** (5.82 g, 35.4 mmol, quantitative conversion was assumed), which was used without further purification for the next step.

1.2.3. 10-Butylbenzo[*g*]pteridine-2,4(3*H*,10*H*)-dione (SI-3)

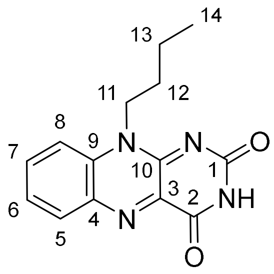

SI-3

Under inert conditions, to the orange solution containing diamine SI-**2** (5.82 g, 35.4 mmol, 1.00 equiv.) in acetic acid (35.4 mL, 1.00 M), boron oxide (9.87 g, 141 mmol, 4.00 equiv.) and alloxane monohydrate (14.2 g, 88.6 mmol, 2.50 equiv.) were added. At room temperature, the solution was stirred for 16 h. After completion of the reaction, the solvent was removed *in vacuo*. Water (100 mL) was added and the reaction flask subjected to sonification. The suspension was filtrated using a vacuum filter. The solid residue was washed with water multiple times to remove excess boron oxide and alloxane. After washing, the residue was dried *in vacuo* affording flavin SI-**3** as orange solid (3.54 g, 13.1 mmol, 37 %). No further purification was performed.

1.2.4. 3,10-Dibutylbenzo[*g*]pteridine-2,4(3*H*,10*H*)-dione (1)

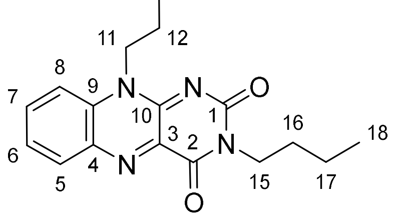

1

Flavin SI-**3** (1.00 g, 3.70 mmol, 1.00 equiv.) was dissolved in *N*,*N*-dimethyl formamide (anhydrous, 37.0 ml, 0.1 M). Potassium carbonate (4.09 g, 29.6 mmol, 8.00 equiv.) was added before dropwise addition of *n*-butyliodide (3.33 ml, 5.39 g, 29.6 mmol, 8.00 equiv.) (**caution:** keep an aqueous ammonia solution nearby to quench the reagent). At room temperature, the yellow solution was stirred for 16 h before adding ammonia (25 % aqueous solution) to quench the excess reagent. The suspension was partitioned between CH_2Cl_2 (200 mL) and brine (200 mL). The aqueous phase was extracted with CH_2Cl_2 (3x 100 mL). All combined organic phases were dried ($MgSO_4$), filtrated, and concentrated *in vacuo*. The crude product was purified *via* column chromatography (silica, CH_2Cl_2/acetone, 100/0 -> 97/3 -> 95/5) to afford alkylated flavin **1**.

Yellow solid (704 mg, 2.15 mmol, 58 %), **TLC:** R_f = 0.41 (CH_2Cl_2/acetone 95/5); **^{1}H NMR** (400 MHz, $CDCl_3$, 298 K): δ (ppm) = 8.32 – 8.25 (m, 1H, H^5), 8.05 – 7.77 (m, 1H, H^8), 7.77 – 7.55 (m, 2H, $H^{6,7}$), 4.81 – 4.64 (m, 2H, H^{11}), 4.17 – 4.01 (m, 2H, H^{15}), 1.88 – 1.77 (m, 2H, H^{12}), 1.75 – 1.64 (m, 2H, H^{16}), 1.61 – 1.50 (m, 2H, H^{13}), 1.47 – 1.35 (m, 2H, H^{17}), 1.01 (t, $^3J_{H-}$ = 7.3 Hz, 3H, H^{14}), 0.94 (t, $^3J_{H-}$ = 7.3 Hz, 3H, H^{18}); **^{13}C{^{1}H} NMR** (101 MHz, $CDCl_3$, 298 K): δ (ppm) = 159.6 ($C^{1/2}$), 155.7 ($C^{1/2}$), 148.9 (C^{10}), 137.3 (C^3), 136.0 (C^4), 135.6 (C^7), 133.5 (C^5), 132.7 (C^9), 126.4 (C^6), 115.2 (C^8), 44.8 (C^{11}), 42.0 (C^{15}), 30.0 (C^{16}), 29.2 (C^{12}), 20.3 ($C^{13/17}$), 20.3 ($C^{13/17}$), 13.9 ($C^{14/18}$), 13.9 ($C^{14/18}$); **HR-MS** (ESI^+): *m/z* = calc. for M = $[C_{18}H_{23}N_4O_2]^+$: 327.1816 ($[M+H]^+$), found: 327.1809; **IR:** (ATR) $\tilde{\nu}$ [cm^{-1}] = 3046, 2962, 2934, 2872, 1709, 1661, 1644 (C=O), 1611, 1586, 1549, 1524, 1512, 1492, 1463, 1424, 1408, 1371, 1337, 1280, 1246, 1217, 1187, 1147, 1109, 1072, 1028, 986, 963, 948, 923, 891, 880, 847, 821, 808, 782, 768, 759, 736, 715, 700.

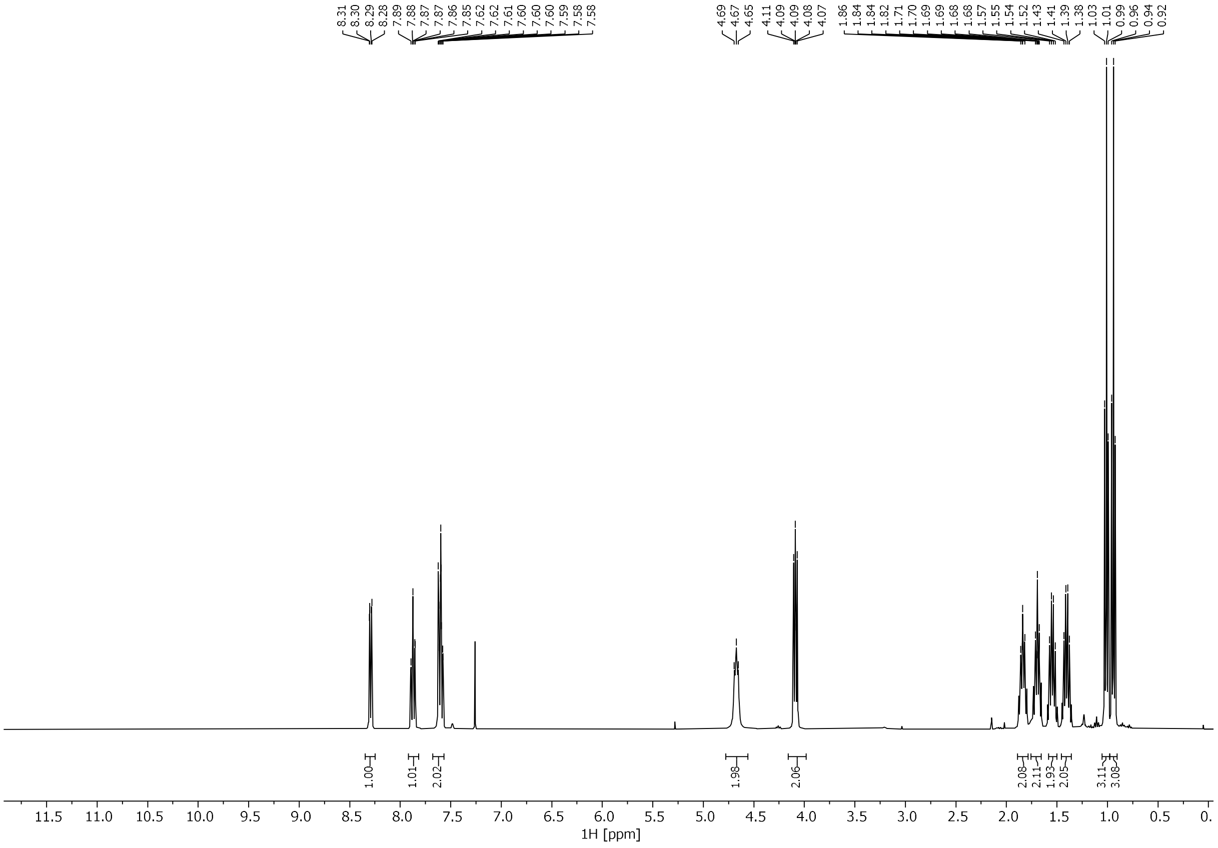

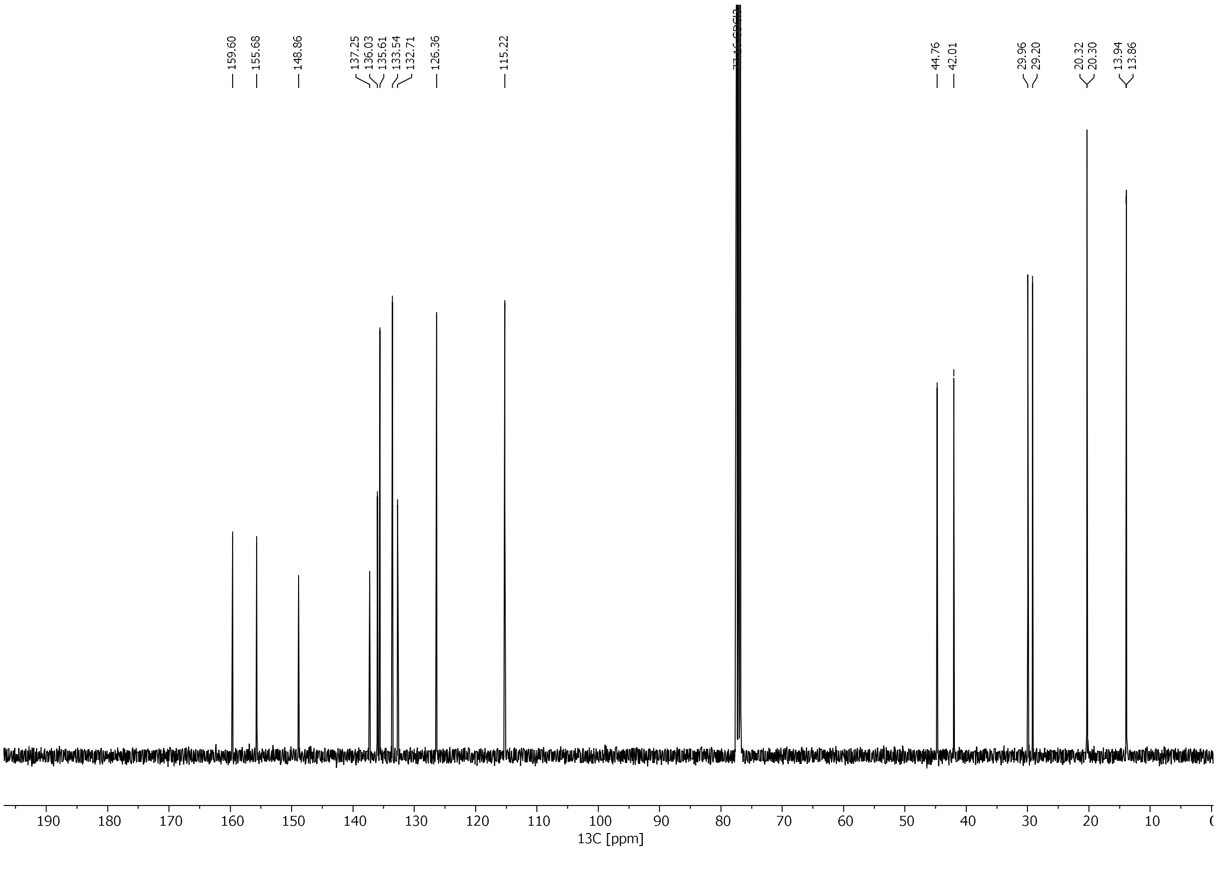

Figure S1 NMR Spectra of flavin **1** ($CDCl_3$, 400 MHz (^1H) and 101 MHz ($^{13}C\{^1H\}$), respectively).

1.3. Transient Absorption Experimental Details

1.3.1. 400 nm pump

The 400 nm excitation pump pulse is obtained via second harmonic generation (SHG) of the 800 nm fundamental pulse inside a 200 μm thin beta-barium borate crystal (BBO, Bluebean Optical Tech Ltd.). Before the BBO, the beam diameter was reduced by a Galilean telescope consisting of a plano-convex lens with 300 mm focal length and a plano-concave lens with 100 mm focal length. The polarization is controlled by a broadband UV (300-470 nm) $\lambda/2$ plate (B.Halle Nachfl. GmbH). The pump pulse with 80 nJ pulse energy is focused using a 250 mm focal length spherical mirror. The pump beam diameter at the sample position is determined to be ~ 150 μm by a beam profiler (CinCam CMOS-1201 CINOGY Tech. GmbH).

1.3.2. NOPA and 325 nm pump

The 325 nm UV-pump is obtained by frequency doubling the output of a tunable non-collinear optical parametric amplifier (NOPA). The NOPA was used to generate 12 nm bandwidth pulses centered at 650 nm, with a resulting pulse duration of 38 fs as determined by Second Harmonic Generation Frequency Resolved Optical Gating (SHG-FROG).

The spectral width is limited by chirping the NOPA pump with a pair of 5 mm fused silica windows post at Brewster angle, and white light with fused silica chirper blocks of 17.88 mm. To increase the SHG efficiency, the 650 nm pulse is focused into the BBO crystal (type I, 40° cut angle, 200 μm) by using a 100 mm focusing spherical mirror and recollimated by a 70 mm spherical focusing mirror. The residual 650 nm fundamental light is filtered out by using a 1 mm thick UG5 filter. The 325 nm UV pump beam is focused with an Al-coated 150 mm focal length spherical focusing mirror to the sample position with a spot size of ~ 150 μm. A ND filter was used to control the 650 nm NOPA output intensity before the prism compressor in an effort to keep UV pulse energies below 60 nJ at the sample position. A broadband (460–680 nm) $\lambda/2$ plate (B.Halle Nachfl. GmbH) is placed before the SHG-BBO to set the polarization. The polarization of the pump pulse is controlled by simultaneously change the orientations of BBO and $\lambda/2$ plate.

1.3.1. CaF_2 white light

White light for detection is generated by focusing 4 µJ of 800 nm light into a 5 mm thick CaF_2 crystal. To avoid damage, the crystal is continuously translated in a plane orthogonal to the beam direction. The focal length is 100 mm and the numerical aperture of the focused beam is chosen to yield a white light spectrum extending down to 370 nm. The white light pulse is collimated using a 100 mm focal length spherical mirror and the intensity of the 800 nm driving pulse is decreased using a heat absorbing filter.

The white light is split into a probe part and a reference part. The probe pulse is focused into the sample by using a 150 mm focal length spherical mirror and the spot size is ~ 30 µm. The probe spatially overlaps with the center of pump and is recollimated after the sample by using an achromatic lens with 75 mm focal length. The probe and reference laser pulses are detected using a home-built prism spectrometer in combination with a pair of high-speed CMOS linear array cameras (Glaz LineScan-I-Gen2, Synertronic Designs).

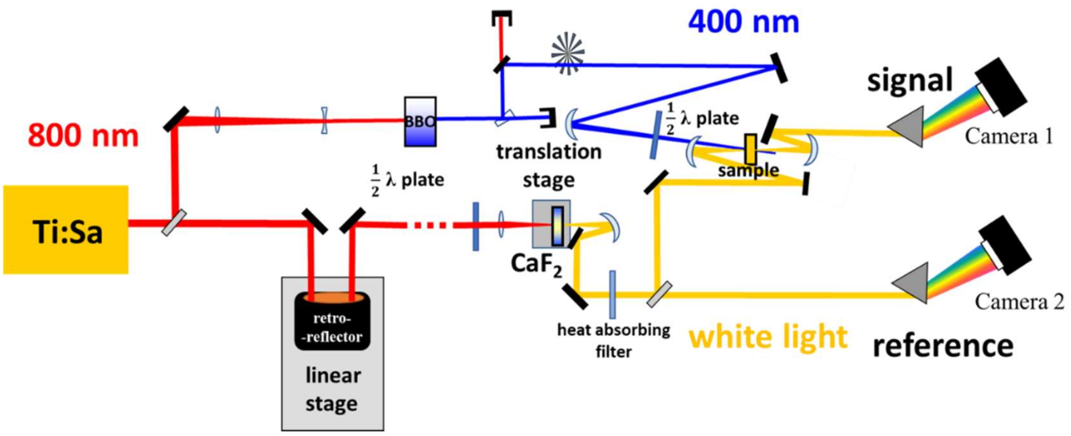

Figure S2 400 nm pumped transient spectroscopy set-up.

172 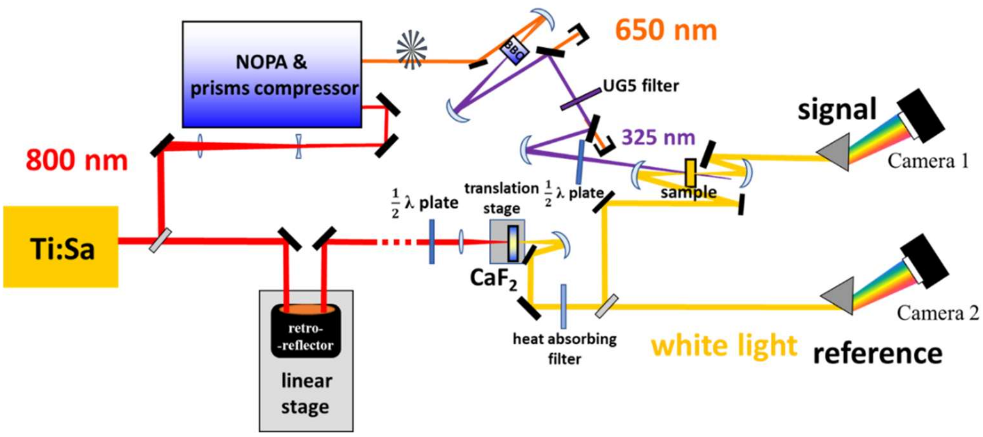

173 Figure S3 325 nm pumped transient spectroscopy set-up.

174

2. Transient Anisotropy Spectra Time Traces

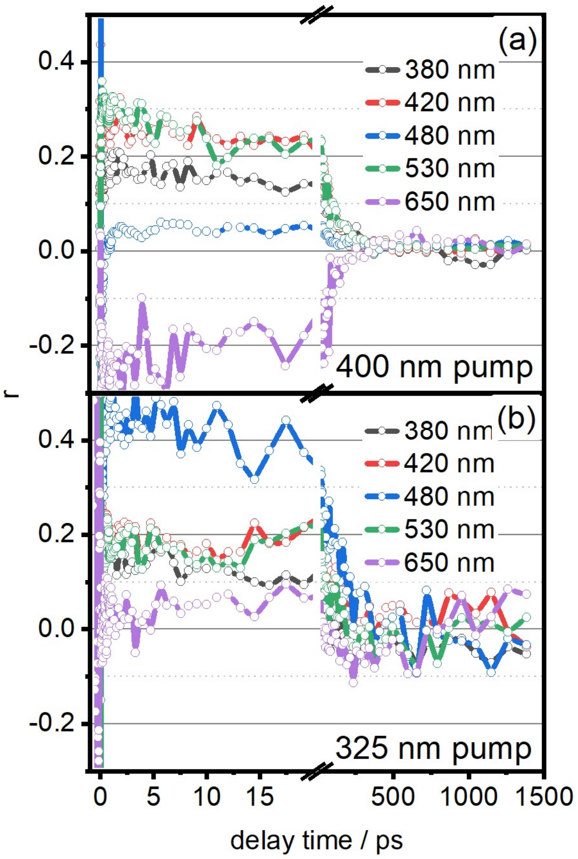

Figure S4 Time traces of transient absorption anisotropies at 380, 420, 480, 530 and 650 nm for (a) 400 nm and (b) 325 nm pumped measurements, the TAA traces indicate no significant rotational diffusion of the sample in 20 ps.

4. Evolution Associated Spectra

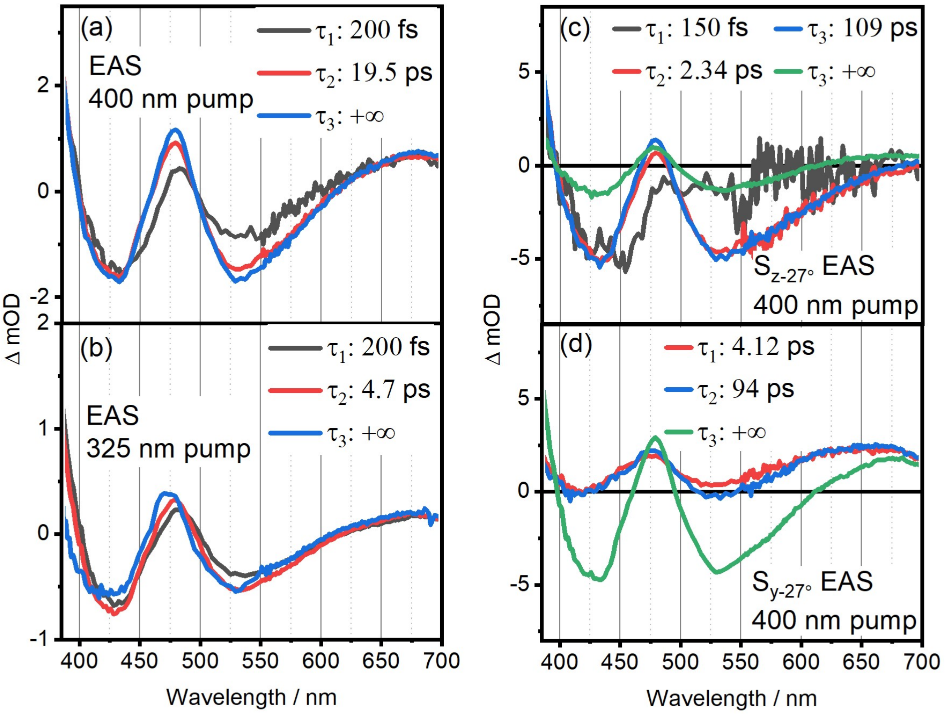

Figure S5 Evolution associated spectra (EAS) from GLA of MA-TA data for **1** with pump wavelength (a) 400 nm and (b) 325 nm, and EAS that for $S_{z-27^\circ}^{400\ nm}$ and $S_{y-27^\circ}^{400\ nm}$ are showing in (c) and (d).

We apply GTA to $S_{z-2\ ^\circ}^{400\ nm}$ and $S_{y-27^\circ}^{400\ nm}$, depicted in Figure S5 (c) and (d), respectively. Similar to TA-MA data, the $S_{z-27^\circ}^{400\ nm}$ dataset displays a <200 fs species dominated by GSB and SE components but this component is not observable in the $S_{y-27^\circ}^{400\ nm}$. However, both datasets contain the few ps species, the ~100 ps species, and long decay time species. The ~100 ps components are related to rotational diffusion and are exclusively found in the datasets based on PAS.

5. Red-ESA disentanglement

Details of Takaya et al.'s method to resolved the spectral components for two overlapping species.[2] By applying eq. S1 and eq. S2 two components with different initial anisotropy values can be isolated:

$$S_{MA}^{(1)}(\lambda,t) = \frac{r^{obs}(\lambda,t)\exp\left(\frac{t}{T_{rot}}\right) - r^{(2)}(\lambda,0)}{r^{(1)}(\lambda,0) - r^{(2)}(\lambda,0)} S_{MA}^{obs}(\lambda,t), \qquad \text{eq. S1}$$

$$S_{MA}^{(2)}(\lambda,t) = \frac{r^{(1)}(\lambda,0) - r^{obs}(\lambda,t)\exp\left(\frac{t}{T_{rot}}\right)}{r^{(1)}(\lambda,0) - r^{(2)}(\lambda,0)} S_{MA}^{obs}(\lambda,t), \qquad \text{eq. S2}$$

where $r^{obs}(\lambda,t)$ is the observed TAA and $S_{MA}^{obs}(\lambda,t)$ is the observed MA spectrum. $r^{(1)}(\lambda,0)$ and $r^{(2)}(\lambda,0)$ are the TAA value of the single transitions and $S_{MA}^{(1)}(\lambda,t)$ and $S_{MA}^{(2)}(\lambda,t)$ are their corresponding isolated MA spectra. T_{rot} is the lifetime of rotational diffusion, which is long compared to our time-window here, meaning that the term $exp\left(\frac{t}{T_{rot}}\right)$ approaches 1. We take TAA values at 550 nm and 700 nm as the TAA for single transitions, and by applying eq. S1 and eq. S2 we obtain the pure SE and pure red-ESA spectra between 550 and 700 nm, see Figure S6 **Fehler! Verweisquelle konnte nicht gefunden werden.** (b). By adding the pure spectra together, we retrieve the MA spectrum (green vs. blue line).

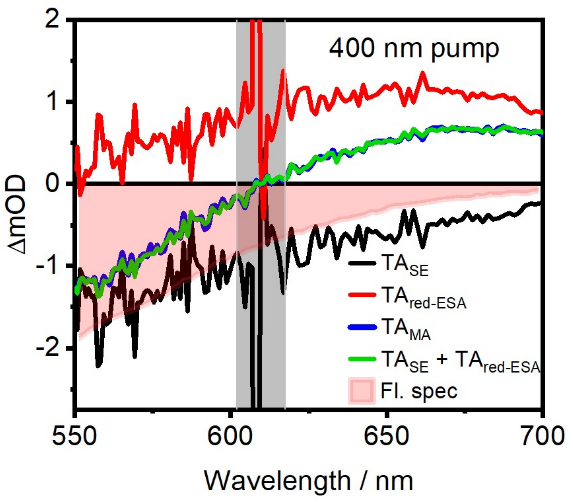

Figure S6 ESA spectra (red solid line) and pure SE signal (black) extracted from the total TA

signal (blue) according to eq. S1 and eq. S2. The wavelength where the MA spectra cross zero are indicated by grey areas

6. PAS for $\beta = 0^{\circ}$ and $\beta = 40^{\circ}$

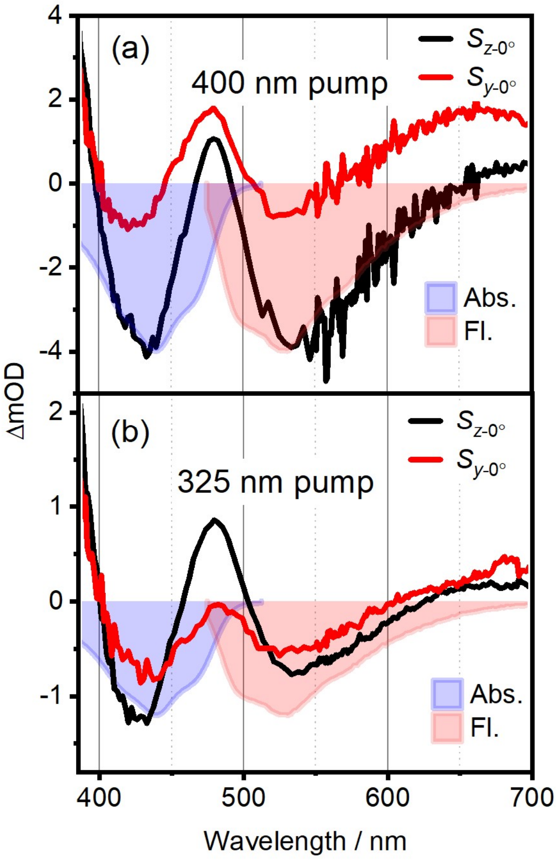

Figure S7 Polarization associated spectra calculation of (a) 400 nm and (b) 325 nm pumped measurements. S_z (black solid) and S_y (red solid) components of with 0° coordinate system rotation, compare with negatively plotted absorption and fluorescence spectra (blue filled and red filled)

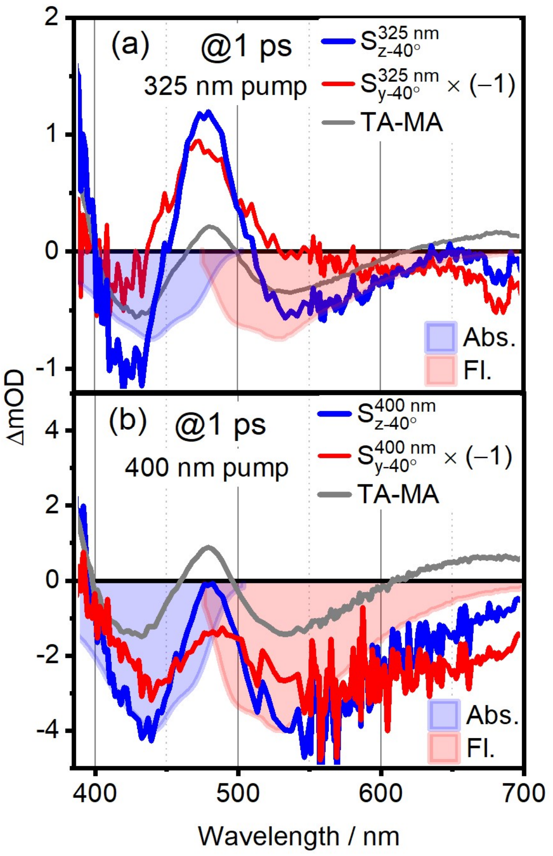

Figure S8 (a) Polarization associated spectra (PAS) for 325 nm excitation. The $S^{325nm}_{z-40°}$ component (blue) optimizes the stimulated emission component with respect to the magic angle TA-spectrum (dark gray). The corresponding $S^{325\,nm}_{y-40°}$ component (red) shows good overlap with the pure ESA spectrum (dotted) and over-emphasizes the red ESA transition peaking at 650. b) shows the PAS for 400 nm excitation and $\beta = 40°$.

7. Minimizing Red-ESA

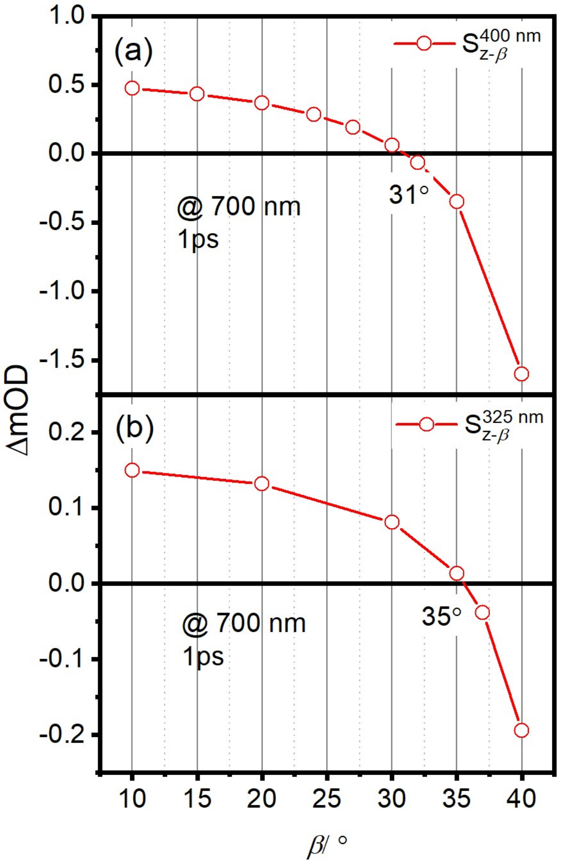

Figure S9 (a) 400 nm and (b) 325 nm pumped S_z signals at 700 nm with the changing of β.

8. Calculation of Palarized Spectra and Anisotropies

Hamm and Zanni[3] give an expression according to *Hochstrasser*[4] for the orientational prefactor of the pump-probe signal depending on the angle between the pumped and probed transition dipole moments and the pump and probe fields. Assuming the molecule has approached a thermal distribution in the S_1 at the time of the probe pulse, this prefactor can be evaluated for each combination of pumped transitions and probed transitions within the molecular ensemble. Here, a single pumped transition corresponds to a transition of one sample of the Wigner distribution in the S_0 minimum to a higher state. A single probed transition corresponds to either A) a transition of one sample of the Wigner distribution in the S_1 minimum to a higher state (excited state absorption) or B) a transition of one sample of the Wigner distribution in the S_1 minimum to a lower state (stimulated emission) or C) a transition of one sample of the Wigner distribution in the S_0 minimum to a higher state (ground state bleach). Rotations and translations of the molecules due to the relaxation in S_1 need to be (approximately) removed prior to the calculation of the spectra (for example by fulfilling the Eckart conditions using a quaternion algorithm).[5-7] Then, the polarized pump probe spectra can be calculated according to:

$$S_{\parallel}(\omega_{pump},\omega_{probe}) \propto \sum_{i}^{\substack{pumped\\transitions}} \sum_{j}^{\substack{probed\\transitions}} (-1)^n g_i(\omega_{pump}) g_j(\omega_{probe}) |\mu_i|^2 |\mu_j|^2 (1 + \frac{2(\mu_i \cdot \mu_j)^2}{|\mu_i|^2 |\mu_j|^2}) \qquad \textit{eq. S3}$$

$$S_{\perp}(\omega_{pump},\omega_{probe}) \propto \sum_{i}^{\substack{pumped\\transitions}} \sum_{j}^{\substack{probed\\transitions}} (-1)^n g_i(\omega_{pump}) g_j(\omega_{probe}) |\mu_i|^2 |\mu_j|^2 (2 - \frac{(\mu_i \cdot \mu_j)^2}{|\mu_i|^2 |\mu_j|^2}) \qquad \textit{eq. S4}$$

Here, μ_i is the transition dipole moment vector of the transition i and g_i is a lineshape function (in our case, a fixed-width Gaussian centered at the transition frequency for transition i). n is odd for ground state bleach or stimulated emission and even for excited state absorption if the typical sign convention for TA spectroscopy is used. From these spectra, anisotropies can be calculated according to eq. 2 in the main manuscript.

9. ORCA[8] Inputs

S_0 geometry optimization and frequency analysis:

```
! wb97x-d3 ma-def2-TZVP CPCM(DMF) AutoAux RIJCOSX OPT FREQ verytightscf
verytightopt

*xyzfile 0 1 geo.xyz
```

S_1 geometry optimization and frequency analysis:

```
! wb97x-d3 ma-def2-TZVP CPCM(dmf) AutoAux RIJCOSX OPT NUMFREQ verytightscf
verytightopt

%tddft
  nroots 10
  iroot 1
  tda true
  maxdim 5
end

*xyzfile 0 1 geo.xyz
```

Excitation Energies at S_0 Minimum:

```
! RI-SCS-wPBEPP86 CPCM(dmf) RIJCOSX verytightscf defgrid3
%MaxCore 90000

%basis
   basis "aug-cc-pVTZ"
   auxJ "aug-cc-pVTZ/JK"
   auxC "aug-cc-pVTZ/C"
end

%tddft
    lrcpcm true
    cpcmeq false
    iroot 1
    tda true
    triplets false
    nroots 20
    dotrans true
end

*xyzfile 0 1 optfreq0.xyz
```

Excitation Energies at S_1 Minimum:

```
! RI-SCS-wPBEPP86 CPCM(dmf) RIJCOSX verytightscf defgrid3
%MaxCore 90000

%basis
   basis "aug-cc-pVTZ"
   auxJ "aug-cc-pVTZ/JK"
```

```
   auxC "aug-cc-pVTZ/C"
end

%tddft
    lrcpcm true
    cpcmeq true
    iroot 1
    tda true
    triplets false
    nroots 20
    dotrans true
end

*xyzfile 0 1 optfreq1.xyz
```

Conical intersection S_3/S_2

```
! wb97x-d3 ma-def2-TZVP CPCM(dmf) AutoAux RIJCOSX CI-OPT NUMFREQ verytightscf

%tddft
  nroots 10
  iroot 2
  jroot 3
  tda true
  maxdim 5
end

*xyzfile 0 1 start2.xyz
```

Conical Intersection S_2/S_1

```
! wb97x-d3 ma-def2-TZVP CPCM(dmf) AutoAux RIJCOSX CI-OPT NUMFREQ verytightscf

%tddft
  nroots 10
  iroot 1
  jroot 2
  tda true
  maxdim 5
end

*xyzfile 0 1 start2.xyz
```

10. Coordinates of Optimized Structures of 3,10-Dimethylbenzo[*g*]pteridine-2,4(3*H*,10*H*)-dione

S_0 Minimum:

```
28
Coordinates from ORCA-job optfreq0
  C   4.23851478459039     -1.08381262468763      0.00058157339000
  C   4.36445075403855      0.31218200016134      0.00102551683880
  C   3.26157445558767      1.13400590829665      0.00077750923815
  C   1.98003541896443      0.56798952727958      0.00005888924302
  C   1.84664512363408     -0.83187151982743     -0.00020375181732
  C   2.99017436249219     -1.64763518901150     -0.00000001401433
  N   0.83671012831972      1.34424457242778     -0.00033940718913
  N   0.61751628670228     -1.42532553567634     -0.00051922468987
  C   -0.42577102918346     -0.67633268988263     -0.00056827477482
  C   -0.39090265675847      0.77109229635780     -0.00058709314992
  C   -1.76605914574389     -1.33412739895975     -0.00041178178854
  N   -1.46346867573980      1.51428624642725     -0.00093794425536
  C   -2.69670012188802      0.93443540481365     -0.00084449480897
  N   -2.82732810960915     -0.46919621573888      0.00037368307788
  O   -3.71570898948508      1.60315809369119     -0.00160052169061
  C   -4.18332985717295     -1.00691432271613      0.00187800142579
  H   5.12367863797869     -1.70672171245055      0.00074725362108
  H   5.35052446989323      0.76062181174973      0.00161089344574
  H   3.39434867531856      2.20582679156154      0.00120184154458
  H   2.84675969041802     -2.72106098318571     -0.00028348917836
  H   -4.71500788078144     -0.67226729076667     -0.88696034406074
  H   -4.71599100579992     -0.66299766071344      0.88649111048473
  H   -4.11722875563099     -2.08944157197428      0.00749234159472
  C   0.96212143793764      2.80096500127656     -0.00041654816073
  H   -0.03180526346773      3.22955881826940     -0.00195471828553
  H   1.50274570832851      3.11831312689894     -0.89098063771696
  H   1.50015199598675      3.11872921528045      0.89158898087409
  O   -1.88836784002982     -2.54157216590090     -0.00074863729737
```

S_1 Minimum:

```
28
Coordinates from ORCA-job optfreq1
  C   4.23851478459039     -1.08381262468763      0.00058157339000
  C   4.36445075403855      0.31218200016134      0.00102551683880
  C   3.26157445558767      1.13400590829665      0.00077750923815
  C   1.98003541896443      0.56798952727958      0.00005888924302
  C   1.84664512363408     -0.83187151982743     -0.00020375181732
  C   2.99017436249219     -1.64763518901150     -0.00000001401433
  N   0.83671012831972      1.34424457242778     -0.00033940718913
  N   0.61751628670228     -1.42532553567634     -0.00051922468987
  C   -0.42577102918346     -0.67633268988263     -0.00056827477482
  C   -0.39090265675847      0.77109229635780     -0.00058709314992
  C   -1.76605914574389     -1.33412739895975     -0.00041178178854
  N   -1.46346867573980      1.51428624642725     -0.00093794425536
  C   -2.69670012188802      0.93443540481365     -0.00084449480897
  N   -2.82732810960915     -0.46919621573888      0.00037368307788
  O   -3.71570898948508      1.60315809369119     -0.00160052169061
  C   -4.18332985717295     -1.00691432271613      0.00187800142579
  H   5.12367863797869     -1.70672171245055      0.00074725362108
  H   5.35052446989323      0.76062181174973      0.00161089344574
  H   3.39434867531856      2.20582679156154      0.00120184154458
  H   2.84675969041802     -2.72106098318571     -0.00028348917836
  H   -4.71500788078144     -0.67226729076667     -0.88696034406074
  H   -4.71599100579992     -0.66299766071344      0.88649111048473
  H   -4.11722875563099     -2.08944157197428      0.00749234159472
  C   0.96212143793764      2.80096500127656     -0.00041654816073
  H   -0.03180526346773      3.22955881826940     -0.00195471828553
  H   1.50274570832851      3.11831312689894     -0.89098063771696
  H   1.50015199598675      3.11872921528045      0.89158898087409
  O   -1.88836784002982     -2.54157216590090     -0.00074863729737
```

S_3/S_2 Conical Intersection

```
28
Coordinates from ORCA-job optfreq_coin32
  C   4.29425661956934     -1.07245811045370      0.01736912472934
  C   4.40913310564715      0.29230111706939      0.07377031700493
  C   3.24114678197731      1.10859761219701      0.05900173763550
  C   1.96465690932745      0.55638637392704     -0.01284559455018
  C   1.82577633538214     -0.84751042214504     -0.05662889960939
  C   3.02125456976781     -1.64316385220482     -0.04373478987359
  N   0.82868532844543      1.34146710810086     -0.03639228518863
  N   0.64906481276311     -1.46028499117089     -0.09036270306285
  C   -0.45433319398412     -0.66952517242481     -0.05228515215354
  C   -0.41474507300613      0.73858513741422     -0.01526732307550
  C   -1.75742402777369     -1.31067061087338     -0.03886704162943
  N   -1.47970648925671      1.51141992558395      0.03089634515704
  C   -2.71218792450388      0.95269386782956      0.04899343702584
  N   -2.83502221562247     -0.44285297902088      0.02024459647610
  O   -3.73698959365906      1.63031851253402      0.09177848897389
  C   -4.18223947543245     -0.99217638268569      0.04633469056959
  H   5.16838197037306     -1.70931752150208      0.02565441582693
  H   5.37700005857043      0.77247908069339      0.12902932496291
  H   3.37086247288016      2.18062326474964      0.11099026824767
  H   2.89595011803139     -2.71914114519348     -0.08296069962496
  H   -4.72796710861203     -0.69701423929859     -0.84971950992877
  H   -4.71644922970621     -0.62151578820991      0.91948454181732
  H   -4.10544075311393     -2.07321187827696      0.08899975928866
```

```
  C   0.94767520081201      2.79090067593374     -0.06774880238264
  H   -0.04467565027463      3.20842455790686     -0.18652237775283
  H   1.57372169780928      3.09081692799728     -0.90865707879818
  H   1.38467406823780      3.15806543733269      0.86301260892652
  O   -1.90677431464855     -2.52810550580942     -0.07709739901175
```

S_2/S_1 Conical Intersection

```
28
Coordinates from ORCA-job optfreq_coin21
  C   4.27991074680356     -1.06375940370183      0.01718803402552
  C   4.39755746703502      0.32554292123023      0.03401887368755
  C   3.27111806979848      1.12878451368743      0.02191415806176
  C   1.99933441922267      0.55961295817400     -0.00518597758850
  C   1.88348046505211     -0.86586681861157     -0.01329701324052
  C   3.03488155507028     -1.65777942031281     -0.00536131788043
  N   0.84017050194790      1.31950213453870     -0.02289480895502
  N   0.64545553114378     -1.37726724051773     -0.02543657716318
  C   -0.47808186662645     -0.69188473018736     -0.01204878774532
  C   -0.39914526271302      0.73676097639180     -0.00552484416589
  C   -1.78396904161621     -1.32407995177701     -0.00607630913656
  N   -1.47124443458215      1.47515212213440      0.01604639732554
  C   -2.72838663518017      0.93663120999243      0.03508708630297
  N   -2.85708983222803     -0.44688260995319      0.00657527679322
  O   -3.72257010659799      1.64565703999968      0.07408251763989
  C   -4.21102432191322     -0.98713325060308      0.00889227758895
  H   5.16798443410037     -1.68309681282262      0.02391974434338
  H   5.37628145912209      0.78718047359585      0.05724436131545
  H   3.38298137493886      2.20366008310632      0.03545604523739
  H   2.91825204194953     -2.73338766829034     -0.01526476173739
  H   -4.78050005530258     -0.54786863517970     -0.80761123108276
  H   -4.70580323640045     -0.75730537744331      0.95208399225045
  H   -4.14458478298816     -2.06227330579782     -0.11889014804996
  C   0.95378424004256      2.77290842982025     -0.05019326607771
  H   -0.04345533743455      3.19215965081560     -0.11368842033801
  H   1.53613399453171      3.07617162291252     -0.92010071453278
  H   1.44313966504219      3.12404588160678      0.85898426625489
  O   -1.92632605221816     -2.53905379280765     -0.01344885313294
```

11. Calculated (Transient-) Absorption Energies and Transition Dipoles at the Minima

S_0 Minimum:

ABSORPTION SPECTRUM VIA TRANSITION ELECTRIC DIPOLE MOMENTS

State	Energy (cm-1)	Wavelength (nm)	fosc	T2 (au**2)	TX (au)	TY (au)	TZ (au)
1	24949.6	400.8	0.500180264	6.59993	-2.52963	-0.44824	-0.00071
2	30453.6	328.4	0.002719401	0.02940	0.00032	0.00021	-0.17146
3	32603.4	306.7	0.172287153	1.73967	1.21262	-0.51887	0.00009
4	33991.8	294.2	0.000054868	0.00053	-0.00057	-0.00007	0.02304
5	37531.7	266.4	0.388345466	3.40640	-1.75621	0.56755	-0.00048
6	39009.2	256.3	0.729034409	6.15257	2.09466	-1.32852	0.00046
7	40311.7	248.1	0.000055871	0.00046	-0.00161	-0.00218	0.02119
8	40975.5	244.0	0.042931185	0.34492	0.58688	0.02221	0.00029
9	41007.5	243.9	0.000020130	0.00016	-0.00587	0.00196	-0.01111
10	50018.2	199.9	0.001091577	0.00718	0.00064	-0.00028	-0.08476
11	44022.6	227.2	0.106277500	0.79477	-0.63449	-0.62626	-0.00026
12	50689.4	197.3	0.232255834	1.50843	-0.79607	-0.93525	-0.00033
13	55090.0	181.5	0.005040349	0.03012	-0.00016	-0.00090	-0.17355
14	51375.8	194.6	0.692333635	4.43642	2.10117	-0.14666	0.00104
15	56176.9	178.0	0.025476159	0.14930	-0.00213	0.00053	0.38638
16	56782.5	176.1	0.006874126	0.03985	-0.09408	-0.17608	0.00003
17	56701.8	176.4	0.000013758	0.00008	0.00020	-0.00015	0.00893
18	45893.8	217.9	0.000079936	0.00057	-0.00005	0.00027	0.02394
19	57826.0	172.9	0.009123355	0.05194	0.00005	0.00047	-0.22790
20	55209.2	181.1	0.108833044	0.64897	-0.17870	0.78552	0.00044

TRANSIENT ABSORPTION SPECTRUM VIA TRANSITION ELECTRIC DIPOLE MOMENTS

State	Energy (cm-1)	Wavelength (nm)	fosc	T2 (au**2)	TX (au)	TY (au)	TZ (au)
2	5504.0	1816.9	0.000020475	0.00122	0.00034	0.00061	0.03499
3	7653.8	1306.5	0.006340807	0.27274	-0.40515	-0.32953	-0.00014
4	9042.3	1105.9	0.000000822	0.00003	0.00090	0.00044	-0.00538
5	12582.2	794.8	0.009010832	0.23577	0.47662	0.09276	0.00011
6	14059.6	711.3	0.038770372	0.90782	0.85674	-0.41693	0.00008
7	15362.1	651.0	0.000115292	0.00247	-0.00349	-0.00691	0.04910
8	16025.9	624.0	0.048446519	0.99521	0.59171	0.80318	0.00058
9	16057.9	622.7	0.000045046	0.00092	-0.00582	-0.00132	0.02980
10	25068.7	398.9	0.005685801	0.07467	-0.00026	0.00039	0.27325
11	19073.0	524.3	0.504370785	8.70575	-2.69995	1.18997	-0.00082
12	25739.8	388.5	0.145905122	1.86612	1.17909	-0.68983	0.00036
13	30140.5	331.8	0.001615335	0.01764	0.00087	-0.00011	0.13283
14	26426.2	378.4	0.066953669	0.83409	-0.90777	-0.10024	-0.00034
15	31227.4	320.2	0.000513310	0.00541	0.00042	-0.00012	-0.07356
16	31833.0	314.1	0.010109780	0.10455	-0.00308	0.32333	0.00010
17	31752.3	314.9	0.000458026	0.00475	0.00006	0.00004	0.06891
18	20944.2	477.5	0.000305899	0.00481	-0.00007	-0.00055	0.06934
19	32876.5	304.2	0.001887150	0.01890	-0.00082	-0.00037	0.13746
20	30259.6	330.5	0.166807338	1.81479	-0.97542	-0.92916	-0.00021

S_1 Minimum:

ABSORPTION SPECTRUM VIA TRANSITION ELECTRIC DIPOLE MOMENTS

State	Energy (cm-1)	Wavelength (nm)	fosc	T2 (au**2)	TX (au)	TY (au)	TZ (au)
1	20000.4	500.0	0.540587228	8.89822	-2.92201	-0.60005	-0.00102
2	28946.3	345.5	0.307790762	3.50056	1.66954	-0.84452	0.00183
3	29769.4	335.9	0.003812264	0.04216	0.01752	-0.00755	-0.20444
4	32033.9	312.2	0.000153990	0.00158	-0.00128	0.00047	0.03976
5	35995.0	277.8	0.564187350	5.16009	-1.99789	1.08098	-0.00028
6	35188.9	284.2	0.597887057	5.59356	1.97683	-1.29835	0.00091
7	37889.0	263.9	0.000035199	0.00031	-0.00088	0.00045	-0.01746
8	39425.8	253.6	0.006761414	0.05646	0.20652	-0.11751	0.00000
9	48503.9	206.2	0.001846388	0.01253	-0.00043	0.00003	0.11195
10	39932.7	250.4	0.000080162	0.00066	0.00328	-0.00061	0.02549
11	42330.9	236.2	0.295297743	2.29656	-1.21365	-0.90753	-0.00056
12	47376.4	211.1	0.348686341	2.42297	1.25748	0.91746	0.00092
13	53540.4	186.8	0.009607262	0.05907	-0.00050	-0.00079	-0.24305
14	51937.3	192.5	0.335139256	2.12433	1.41662	-0.34279	0.00129
15	54776.8	182.6	0.022783270	0.13693	-0.00305	0.00047	0.37003
16	54073.4	184.9	0.048954934	0.29805	-0.51386	-0.18437	-0.00044
17	51749.3	193.2	0.170308478	1.08345	-0.39650	0.96241	0.00121
18	52193.7	191.6	0.243810153	1.53783	-0.00847	1.24007	0.00064
19	44859.8	222.9	0.000129268	0.00095	-0.00051	0.00225	-0.03071
20	56620.6	176.6	0.007010349	0.04076	-0.00003	-0.00064	-0.20189

TRANSIENT ABSORPTION SPECTRUM VIA TRANSITION ELECTRIC DIPOLE MOMENTS

State	Energy (cm-1)	Wavelength (nm)	fosc	T2 (au**2)	TX (au)	TY (au)	TZ (au)
2	8945.9	1117.8	0.017932463	0.65992	-0.72188	-0.37256	-0.00072
3	9769.0	1023.6	0.000068735	0.00232	-0.00636	-0.00258	0.04764
4	12033.5	831.0	0.000006197	0.00017	0.00161	0.00046	-0.01291
5	15994.6	625.2	0.008234638	0.16949	0.41076	0.02774	-0.00019
6	15188.6	658.4	0.058215964	1.26183	1.07380	-0.32984	0.00026
7	17888.6	559.0	0.000168988	0.00311	0.00061	-0.00313	-0.05568
8	19425.4	514.8	0.062071193	1.05195	-0.09012	1.02168	-0.00044
9	28503.5	350.8	0.005571161	0.06435	0.00088	-0.00091	-0.25366
10	19932.3	501.7	0.000058597	0.00097	0.00557	-0.00001	-0.03061
11	22330.5	447.8	0.444871535	6.55860	-2.43277	0.80015	-0.00089
12	27376.0	365.3	0.213828544	2.57141	-1.38339	0.81095	-0.00056
13	33540.0	298.2	0.001328237	0.01304	0.00063	0.00048	0.11418
14	31936.9	313.1	0.085369054	0.88000	-0.93737	0.03669	-0.00039
15	34776.5	287.6	0.000047347	0.00045	0.00221	-0.00029	-0.02105
16	34073.0	293.5	0.092250758	0.89132	0.70654	0.62620	0.00027
17	31748.9	315.0	0.143654101	1.48958	-0.89338	-0.83154	-0.00021
18	32193.3	310.6	0.066280277	0.67779	0.76863	0.29496	0.00014
19	24859.4	402.3	0.000373425	0.00495	0.00206	0.00099	-0.07029
20	36620.2	273.1	0.000782748	0.00704	-0.00157	-0.00079	0.08387

References

(1) Fulmer, G. R.; Miller, A. J.; Sherden, N. H.; Gottlieb, H. E.; Nudelman, A.; Stoltz, B. M.; Bercaw, J. E.; Goldberg, K. I., NMR chemical shifts of trace impurities: common laboratory solvents, organics, and gases in deuterated solvents relevant to the organometallic chemist. *Organometallics* **2010,** *29* (9), 2176-2179.

(2) Takaya, T.; Hamaguchi, H.-o.; Iwata, K., Femtosecond time-resolved absorption anisotropy spectroscopy on 9, 9 ´ -bianthryl: Detection of partial intramolecular charge transfer in polar and nonpolar solvents. *J. Chem. Phys.* **2009,** *130* (1).

(3) Hamm, P.; Zanni, M., *Concepts and methods of 2D infrared spectroscopy*. Cambridge University Press: 2011.

(4) Hochstrasser, R. M., Two-dimensional IR-spectroscopy: polarization anisotropy effects. *Chem. Phys.* **2001,** *266* (2-3), 273-284.

(5) Dymarsky, A. Y.; Kudin, K. N., Computation of the pseudorotation matrix to satisfy the Eckart axis conditions. *J. Chem. Phys.* **2005,** *122* (12).

(6) Coutsias, E. A.; Seok, C.; Dill, K. A., Using quaternions to calculate RMSD. *J. Comput. Chem.* **2004,** *25* (15), 1849-1857.

(7) Kudin, K. N.; Dymarsky, A. Y., Eckart axis conditions and the minimization of the root-mean-square deviation: Two closely related problems. *J. Chem. Phys.* **2005,** *122* (22).

(8) Neese, F., Software update: The ORCA program system—Version 5.0. *WIREs Comput. Mol. Sci.* **2022,** *12* (5), e1606.
